# GRK5 is a regulator of fibroblast activation and cardiac fibrosis

**DOI:** 10.1073/pnas.2012854118

**Published:** 2021-01-26

**Authors:** Akito Eguchi, Ryan Coleman, Kenneth Gresham, Erhe Gao, Jessica Ibetti, J. Kurt Chuprun, Walter J. Koch

**Affiliations:** ^a^Center for Translational Medicine, Lewis Katz School of Medicine at Temple University, Philadelphia, PA 19140;; ^b^Department of Pharmacology, Lewis Katz School of Medicine at Temple University, Philadelphia, PA 19140

**Keywords:** fibrosis, GRK5, heart failure

## Abstract

Pathological remodeling of the heart is a hallmark of chronic heart failure (HF) and these structural changes further perpetuate the disease. Cardiac fibroblasts are the critical cell type that is responsible for maintaining the structural integrity of the heart. Stress conditions, such as a myocardial infarction (MI), can activate quiescent fibroblasts into synthetic and contractile myofibroblasts. G protein-coupled receptor kinase 5 (GRK5) is an important mediator of cardiovascular homeostasis through dampening of GPCR signaling, and is expressed in the heart and up-regulated in human HF. Of note, GRK5 has been demonstrated to translocate to the nucleus in cardiomyocytes in a calcium-calmodulin (Ca^2+^-CAM)-dependent manner, promoting hypertrophic gene transcription through activation of nuclear factor of activated T cells (NFAT). Interestingly, NFAT is also involved in fibroblast activation. GRK5 is highly expressed and active in cardiac fibroblasts; however, its pathophysiological role in these crucial cardiac cells is unknown. We demonstrate using adult cardiac fibroblasts that genetic deletion of GRK5 inhibits angiotensin II (AngII)-mediated fibroblast activation. Fibroblast-specific deletion of GRK5 in mice led to decreased fibrosis and cardiac hypertrophy after chronic AngII infusion or after ischemic injury compared to nontransgenic littermate controls (NLCs). Mechanistically, we show that nuclear translocation of GRK5 is involved in fibroblast activation. These data demonstrate that GRK5 is a regulator of fibroblast activation in vitro and cardiac fibrosis in vivo. This adds to previously published data which demonstrate the potential beneficial effects of GRK5 inhibition in the context of cardiac disease.

## Significance

Pathological remodeling of the heart is a hallmark of chronic heart failure (HF) and these structural changes further perpetuate the disease. G protein-coupled receptor (GPCR) kinase 5 (GRK5) has been shown to cause deleterious effects on the cardiomyocyte during HF; however, its effects in cardiac fibroblasts, the crucial cell type responsible for maintaining the structural integrity of the heart, is not understood. Here, we use in vitro and in vivo methods to demonstrate that inhibition of GRK5 inhibits fibroblast activation and attenuates the fibrotic response in the heart.

Heart failure (HF) is a clinical syndrome characterized by the heart’s inability to pump blood sufficiently to meet the metabolic demands of the body. To combat the decrease in cardiac function, various compensatory mechanisms are activated to maintain perfusion. The renin-angiotensin-aldosterone system (RAAS) and sympathetic nervous system (SNS) activate in order to restore lost cardiac output following cardiac injury, such as myocardial infarction (MI) (1–3). However, sustained activation of these systems exacerbates the condition, causing hemodynamic stress and myocyte death, prompting cardiac remodeling. Cardiac remodeling is a major driving force in the development and progression of HF that includes cardiac hypertrophy, ventricular dilation, and fibrosis (4–6). The interstitial fibrosis, which decreases compliance as well as promotes arrhythmogenesis, is due to excessive extracellular matrix proteins deposited by pathologically activated fibroblasts called myofibroblasts. After stress, the quiescent fibroblasts transdifferentiate into myofibroblasts, which have de novo expression of α-smooth muscle actin (αSMA), as well as hypersecrete extracellular matrix proteins (7, 8).

During acute tissue injury, profibrotic cytokines such as angiotensin II (AngII) and transforming growth factor β (TGFβ) are released by inflammatory and mesenchymal cells, which leads to the activation of fibroblasts (7, 9). AngII signals through angiotensin type I receptor (AT1R), a G protein-coupled receptor (GPCR), coupled to the heterotrimeric G protein, Gαq (10). AngII-AT1R signaling promotes fibroblast transdifferentiation through activation of calcium (Ca^2+^) signaling and subsequent gene transcription, primarily through the nuclear factor of activated T cells (NFAT) as well as release of TGFβ and subsequent autocrine TGFβ signaling (7, 10, 11). Pharmacological inhibition of AngII in patients with HF has shown to reduce or delay the fibrotic remodeling of the heart (12).

GPCR kinases (GRKs) are a family of serine threonine protein kinases, which canonically recognize and phosphorylate agonist-activated GPCRs in order to terminate signaling. This is the initiation step in the desensitization process, which also involves β-arrestin binding and GPCR internalization (1–3). GRK5 is a major GRK isoform expressed in the heart and it has been shown to be up-regulated in the myocardium of HF patients (13). GRK5 is a member of the GRK4 subfamily of GRKs, all of which contain a nuclear localization signal (3, 14). In cardiomyocytes, GRK5 translocation to the nucleus has been shown to be crucial for pathological gene transcription following hypertrophic stress and this occurs via noncanonical actions (15–18). This occurs in myocytes downstream of Gαq activation that can occur through selective hypertrophic stressors, including α1-adrenergic and AngII-mediated signaling pathways (16). The gene transcription facilitation induced by nuclear GRK5 in myocytes include NFAT (18).

Novel drug classes for HF treatment have been sparse, and targeting nonmyocytes is a potential alternative approach in the treatment of the disease, especially the cardiac fibroblast. The injured heart contains a variety of signals which promote the transdifferentiation of fibroblasts into myofibroblasts (9, 19). Our incomplete understanding of these regulatory pathways that promote fibroblast activation has prevented the discovery of novel therapies to target the fibrotic response, which could affect many HF conditions, including HF with preserved ejection fraction. Interestingly, GRK5 is highly expressed in the cardiac fibroblast and thus, the current study targets this kinase in cardiac fibroblast function in vitro and in vivo and its role in cardiac fibrosis after injury.

## Methods

Please refer to *SI Appendix* for full detailed materials and methods.

### Experimental Animals.

To obtain inducible, fibroblast-specific GRK5 knockout (KO) mice, collagen1α2- CreER(T) mice (The Jackson Laboratory, stock no. 029567) were crossed with GRK5^fl/fl^ mice (The Jackson Laboratory, stock no. 010960). All animal studies were conducted with the approval of the Animal Care and Use Committee at Temple University.

### Isolation of Adult and Neonatal Mouse Cardiac Fibroblasts and Myocytes.

Hearts were removed from 2- to 3-mo-old mice and adult cardiac fibroblasts (MACFs) and myocytes were isolated as previously described (20). Neonatal rat cardiac fibroblasts (NRCFs) were isolated as a byproduct of neonatal rat cardiac myocyte isolation, performed as previously described (21).

### In Vivo AngII Infusion and Model of Myocardial Infarction.

AngII (1 μg/kg/min) dissolved in phosphate-buffered saline (PBS) was continuously infused subcutaneously into mice via an osmotic minipump (ALZET) for 4 wk. A control group was infused only with PBS.

For our MI model, mice were subjected to permanent ligation of the left main descending coronary artery or a sham surgery as we have described previously and tissue was collected 4 wk post MI (22).

### Assessment of Myocardial Fibrosis and Hypertrophy.

Collagen levels were measured using the Masson’s trichrome staining kit (Sigma HT15) without modifications as previously described (23). For each area of the heart, at least 10 random fields were measured. Images were quantified using CellProfiler, a cell image analysis software, capable of determining fibrotic area in an unbiased manner (24).

Cardiomyocyte hypertrophy was measured using wheat germ agglutinin (WGA) staining as previously described (25).

### Drug Treatments.

Application of recombinant AngII (1 to 10 μM), TGFβ (1 to 10 ng/mL), and ET-1 (100 nM) for 48 h was used to induce myofibroblast transdifferentiation. Nuclear translocation inhibitor malbrancheamide (malb) was used at 1 μM 24 h before AngII treatments (26).

### NFAT Luciferase Reporter and Luciferase Assay.

Cardiac fibroblasts were infected with an NFAT reporter adenovirus at a multiplicity of infection (MOI) of 10. Medium was changed after 24 h, and 48 h after infection, cells were stimulated with AngII for 24 h. Cells were lysed and luciferase activity was measured (11).

### Collagen Gel Contraction Assay.

Fibroblasts were harvested from a confluent monolayer by trypsin-disodium ethylenediaminetetraacetic acid (EDTA) digestion, pelleted, and resuspended in serum-free DMEM. Fibroblasts were then seeded into collagen matrices (0.85 mg/mL) such that each gel contained 100,000 fibroblasts and cast in 24-well plates. The collagen gels were released from the edges, floating in serum-free Dulbecco's Modified Eagle Medium (DMEM) with or without AngII. ImageJ software was used to calculate the surface area, which is reported as values normalized to the initial size of the gel (11).

### Statistical Tests.

Data are expressed as mean ± SD. Statistical significance was determined by ANOVA and Tukey’s multiple comparisons test for multivariate experiments and *t* test for experiments with two groups.

## Results

### GRK5 Is Required for Fibroblast to Myofibroblast Transdifferentiation.

**Fig. 1.**
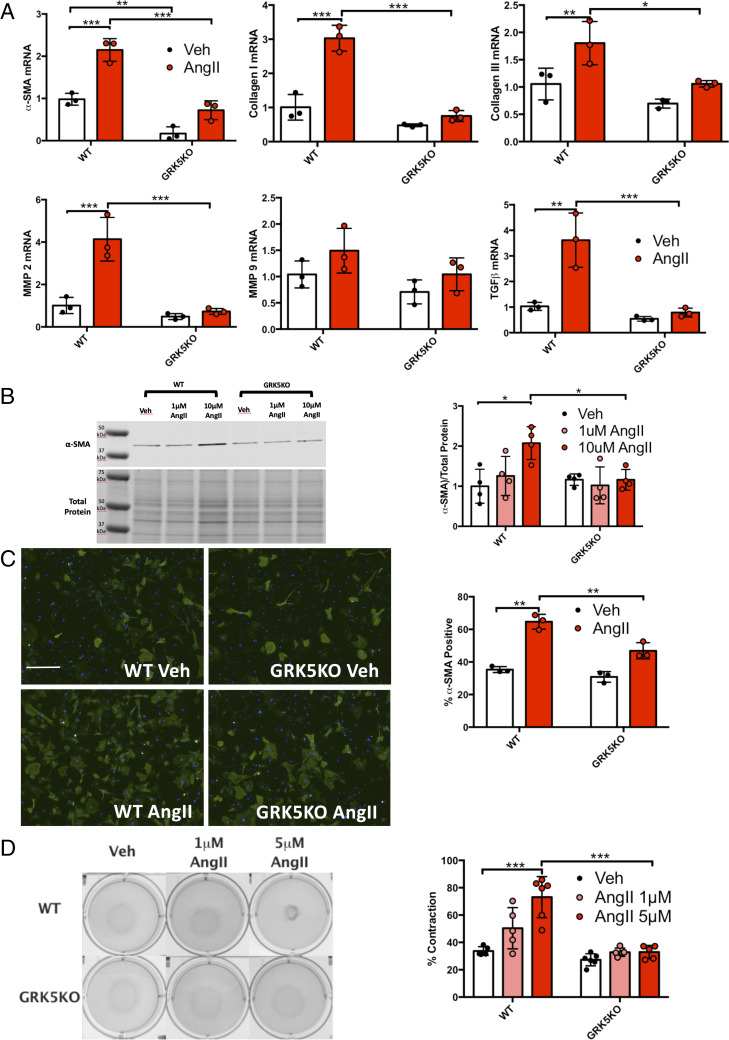
GRK5 is necessary for fibroblast transactivation into myofibroblasts. (*A*) Myofibroblast gene expression in WT and GRK5KO MACFs as measured by qRT-PCR normalized to transcriptionally controlled tumor protein 1 (TPT1); fold change vs. WT Veh. MACFs were stimulated with 1 μM AngII for 48 h. *n* = 3 per group. (*B*) Immunoblot and quantification for α-SMA expression in WT and GRK5KO MACFs stimulated with AngII (normalized to total protein). *n* = 3 per group. (*C*) Immunofluorescent staining and quantification of α-SMA (green)-positive cells in WT and GRK5KO MACFs stimulated with AngII. Cells were counterstained with DAPI. *n* = 3 per group, five images per biological replicate. (*D*) Photographs and quantification of floating collagen gel matrices seeded with WT or GRK5KO MACFs after 18 h of AngII stimulation. *n* = 5 per group. *P ≤ 0.05, **P ≤ 0.01, ***P ≤ 0.001.

To investigate if GRK5 is required for fibroblast activation, we first isolated primary MACFs from wild type (WT) and global GRK5KO mice for in vitro analysis (*SI Appendix*, Fig. S1) (27). WT and GRK5KO MACFs were then treated with AngII for 48 h and qRT-PCR was performed. AngII stimulation caused an up-regulation of many myofibroblast-associated genes in WT MACFs while AngII did not increase expression of α-SMA, collagen I, collagen III, MMP2, and TGFβ in GRK5KO MACFs. (Fig. 1*A*). Further, the AngII-induced expression of α-SMA protein in WT MACFs was not seen after AngII treatment of GRK5KO MACFs (Fig. 1*B*). Immunofluorescence staining for α-SMA stress fiber formation was also inhibited in GRK5KO MACFs compared to WT controls after AngII treatment (Fig. 1*C*). We did not observe any morphological differences between WT and GRK5KO fibroblasts after AngII stimulation. Functionally, the expression of α-SMA fibers in myofibroblasts acts to contract wounds. WT MACFs seeded into a floated collagen matrix demonstrated contraction after AngII stimulation while GRK5KO MACFs did not (Fig. 1*D*). GRK5KO MACFs also demonstrated decreased wound closure as shown by an in vitro scratch assay (*SI Appendix*, Fig. S2). The contraction of collagen gel matrices and scratch assay wound closure was not due to altered MACF proliferation in WT vs. GRK5KO cells (*SI Appendix*, Fig. S3). Finally, myofibroblasts are characterized by their hypersecretory state. It is currently unknown whether activation of fibroblasts is associated with an increase in protein translation. To determine the rate of protein translation, we performed a surface sensing of translation (SUnSET) assay. After 4 h of AngII stimulation, there was a significant increase in puromycin incorporation into newly synthesized proteins in WT MACFs while GRK5KO MACFs had a significantly reduced rate of protein translation, suggesting a more quiescent and fibroblast-like state (*SI Appendix*, Fig. S4).

### Decreased Levels of GRK5 in Fibroblasts Are Beneficial to AngII-Infused Myocardium.

The observation that GRK5 was essential for fibroblast activation in vitro suggested that this kinase might participate in the fibrotic response in the heart in vivo. In order to determine the requirement of GRK5 in myofibroblast transdifferentiation and fibrotic response in vivo, we generated a tamoxifen-inducible, fibroblast-specific, GRK5 KO mouse. These mice were created by crossing mice expressing cre recombinase driven by the Col1a2 enhancer element and GRK5 flox mice (28). Eight-week-old adult male Col1a2-cre/GRK5^fl/fl^ mice and their control littermates were injected intraperitoneally with tamoxifen (100 mg/kg per day) for 5 d to induce a loss of GRK5 in fibroblasts (GRK5 fibroKO). After a 2-wk washout period, freshly isolated MACFs from GRK5 fibroKO mice demonstrated ∼80% GRK5 protein loss compared to MACFs isolated from WT mice (*SI Appendix*, Fig. S5).

**Fig. 2.**
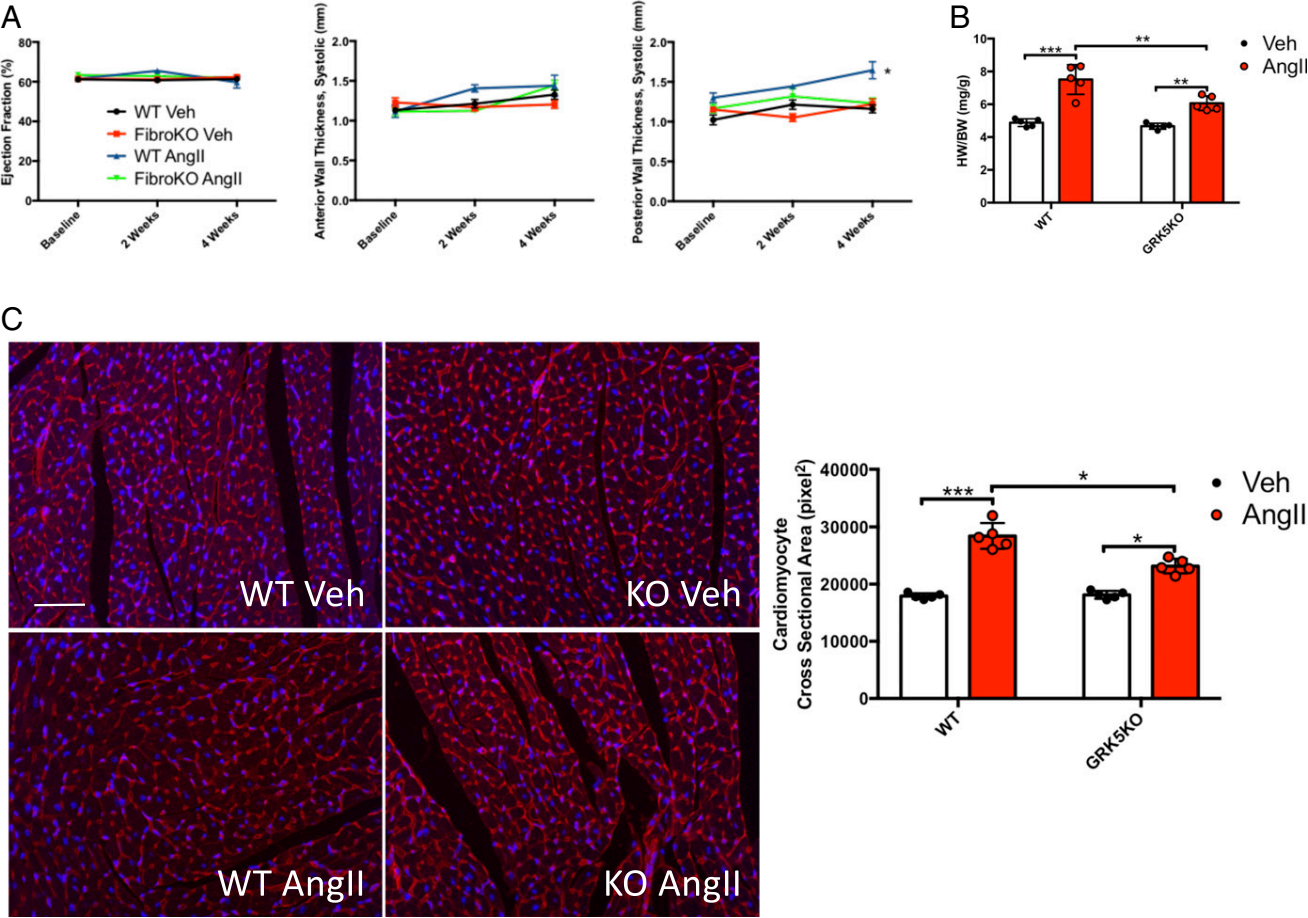
GRK5 fibroKOs are protected against AngII-mediated cardiac hypertrophy. (*A*) Echocardiographic analysis of WT and GRK5 fibroKO mice infused with 1 µg/kg/min with AngII for 4 wk compared to 4 wk of saline infusion. Cardiac function shown by LV ejection fraction and LV wall thickness shown by anterior wall and posterior wall thicknesses at systole. *n* = 5 per group. (*B*) Quantification of heart weight (HW) normalized to body weight (BW) 4 wk after AngII infusion. *n* = 5 per group. (*C*) Representative images and quantification of WGA- and DAPI-stained murine heart sections from hearts 4 wk after AngII infusion. *n* = 5 per group, five images per biological replicate. *P ≤ 0.05, **P ≤ 0.01, ***P ≤ 0.001.

To assess the effects of in vivo GRK5 KO on cardiac fibrosis, WT and GRK5 fibroKO mice were subjected to 4 wk of AngII (1 μg/kg/min) or saline (Veh) for 4 wk. This dose has previously been shown to induce cardiac hypertrophy and fibrosis, without changes in systolic function (29–32). Mice were followed at 2 wk and 4 wk after AngII infusion with echocardiographic assessment of left ventricular (LV) structure and function. Heart rate was unchanged in both WT and GRK5 fibroKO mice after AngII infusion at 4 wk (WT saline 466 ± 30.6; WT AngII 456 ± 19.7; fibroKO saline 452 ± 30.8; fibroKO AngII 450 ± 36.3). Systolic function as measured by LV ejection fraction also remained unchanged as was LV anterior wall dimension at systole (Fig. 2*A*). WT mice infused with AngII demonstrated significantly increased LV posterior wall dimension at systole, while this increase was absent in GRK5 fibroKO mice (Fig. 2*A*). Similar effects were seen during diastole (*SI Appendix*, Fig. S6). We also examined the heart and body weights (HW/BW ratio) of WT and GRK5 fibroKO mice after AngII or Veh treatment. Both WT and GRK5 fibroKO mice had significantly higher heart weights after AngII compared to their respective saline-infused controls; however, GRK5 fibroKO mice had significantly less hypertrophy (Fig. 2*B*).

**Fig. 3.**
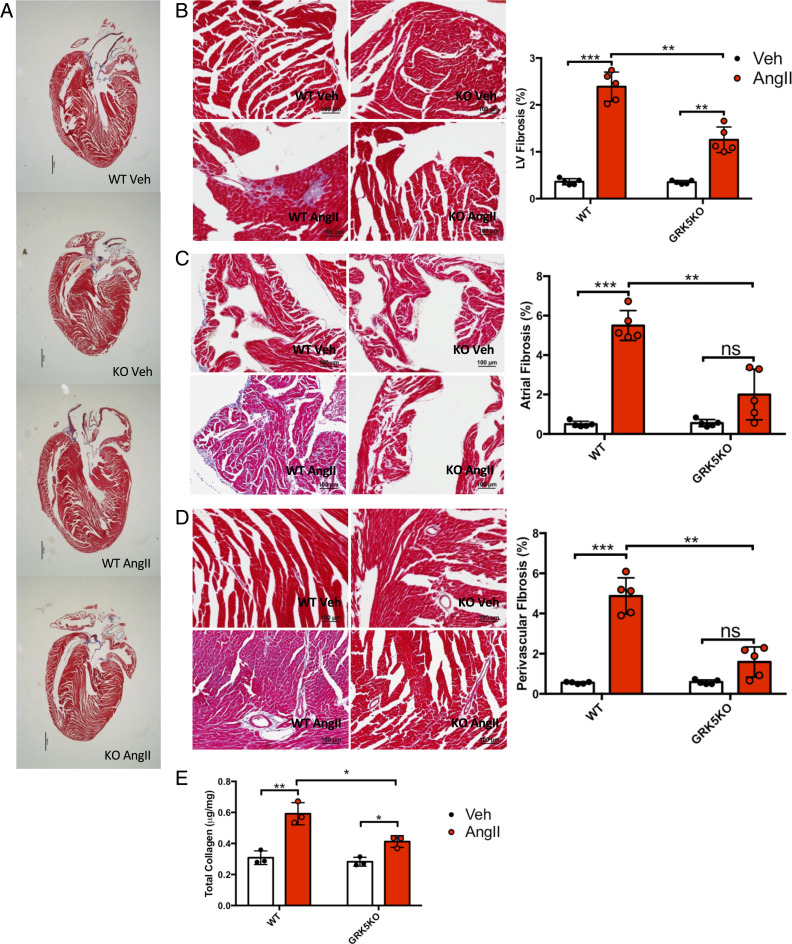
GRK5 fibroKOs are protected against AngII-mediated cardiac fibrosis. (*A*) Whole heart long-axis images of Masson’s trichrome staining. (*B*–*D*) Representative Masson’s trichrome images and quantification of LV (*B*), atria (*C*), and perivascular (*D*) fibrosis after 4 wk of AngII infusion. Quantification expressed as a percentage of fibrosis from the total area. *n* = 5 per group, five images per biological replicate. (*E*) Quantification of collagen in LV lysates using Sirius red collagen quantification kit. *n* = 3 per group. *P ≤ 0.05, **P ≤ 0.01, ***P ≤ 0.001.

We performed histological analysis of both WT and GRK5 fibroKO hearts after 4 wk of AngII infusion and compared these to the corresponding mice treated with saline (Veh). Wheat germ agglutinin staining of the plasma membrane demonstrated increased cardiomyocyte cross-sectional area in both WT and GRK5 fibroKO hearts after AngII infusion compared to their respective saline controls; however, like the HW/BW ratios, GRK5 fibroKO cardiomyocytes had significantly attenuated hypertrophy compared to WT (Fig. 2*C*). AngII induces cardiac fibrosis through activation of fibroblasts. In order to determine the fibrotic response, we utilized Masson’s trichrome staining to stain fibrotic areas within the myocardium (Fig. 3*A*). WT mice demonstrated an increase in fibrosis in the LV, atria, and perivascular regions after 4 wk of AngII while GRK5 fibroKO mice only had a significant increase in LV fibrosis (Fig. 3 *B*–*D*). However, GRK5 fibroKO mice had attenuated fibrosis compared to WT mice in all three regions. Furthermore, we utilized Sirius red to quantify the amount of collagen deposited in the heart after AngII infusion. WT mice demonstrated an increase in collagen content after AngII infusion that was inhibited in our GRK5 fibroKO mice (Fig. 3*E*). Thus, the loss of GRK5 in fibroblasts in vivo protected against AngII-mediated cardiac fibrosis.

### Fibroblast-Specific Loss of GRK5 Protects Against Cardiac Ischemic Injury.

**Fig. 4.**
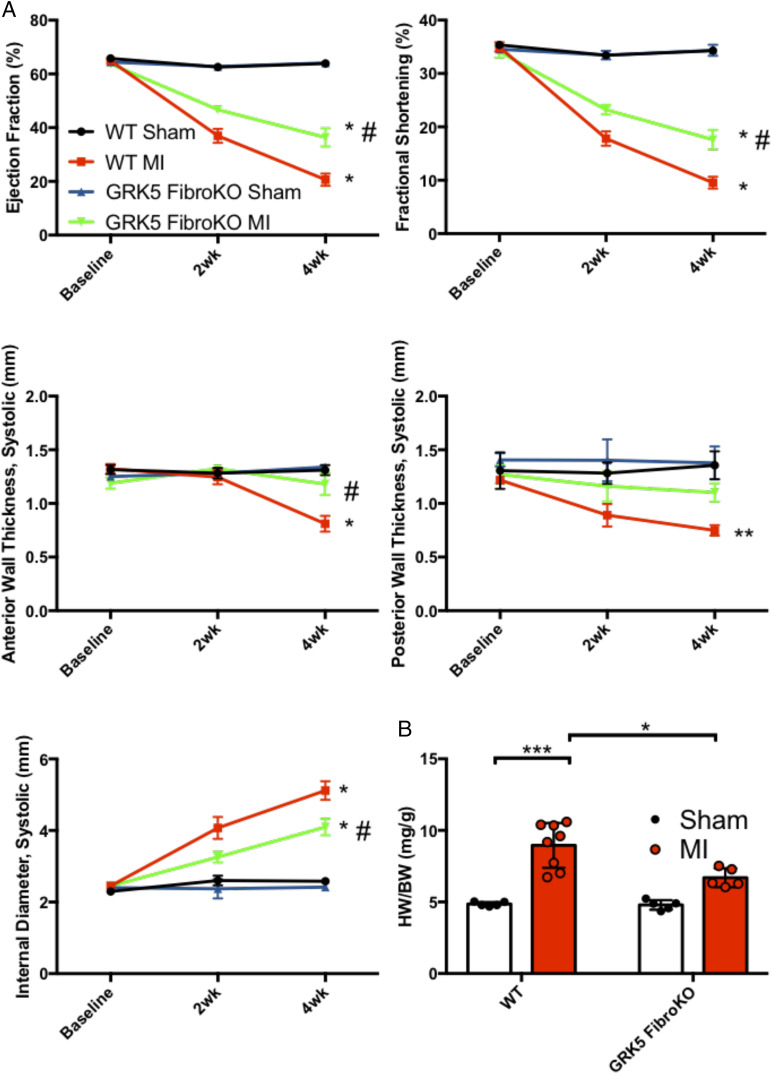
GRK5 fibroKOs are protected against MI mediated cardiac dysfunction and hypertrophy. (*A*) Echocardiographic analysis of WT and GRK5 fibroKO mice that underwent MI surgery. Cardiac function shown by LV ejection fraction and fractional shortening (*Top*). Wall thickness shown by LV anterior wall (LVAW) and posterior wall (LVPW) thicknesses at systole (*Middle*). LV dilation shown by internal diameter. *n* = 5 for sham; 5 to 8 for MI. (*B*) Quantification of HW normalized to BW 4 wk after MI surgery. *n* = 5 for sham; 5 to 8 for MI. *P ≤ 0.05 vs. sham, **P ≤ 0.01, ***P ≤ 0.001, ^#^*P* ≤ 0.05 vs. WT MI.

In order to determine the effect of fibroblast-specific GRK5 knockdown in a more clinically relevant model, we employed a mouse model of MI injury by permanently occluding the coronary artery (22). In vivo characterization was done by performing echocardiography on WT and GRK5 fibroKO mice before and after MI. Control mice presented with decreased LV ejection fraction and LV fractional shortening at 2 wk post-MI that worsened at 4 wk (Fig. 4*A*). However, GRK5 fibroKO mice displayed partial but significantly improved systolic function compared to WT mice after MI (Fig. 4*A*). GRK5 fibroKO mice also demonstrated preserved anterior and posterior wall thickness at systole and diastole as well as LV internal diameter compared to WT mice (Fig. 4*A* and *SI Appendix*, Fig. S7). There were no survival differences between the MI groups 4 wk post-MI.

**Fig. 5.**
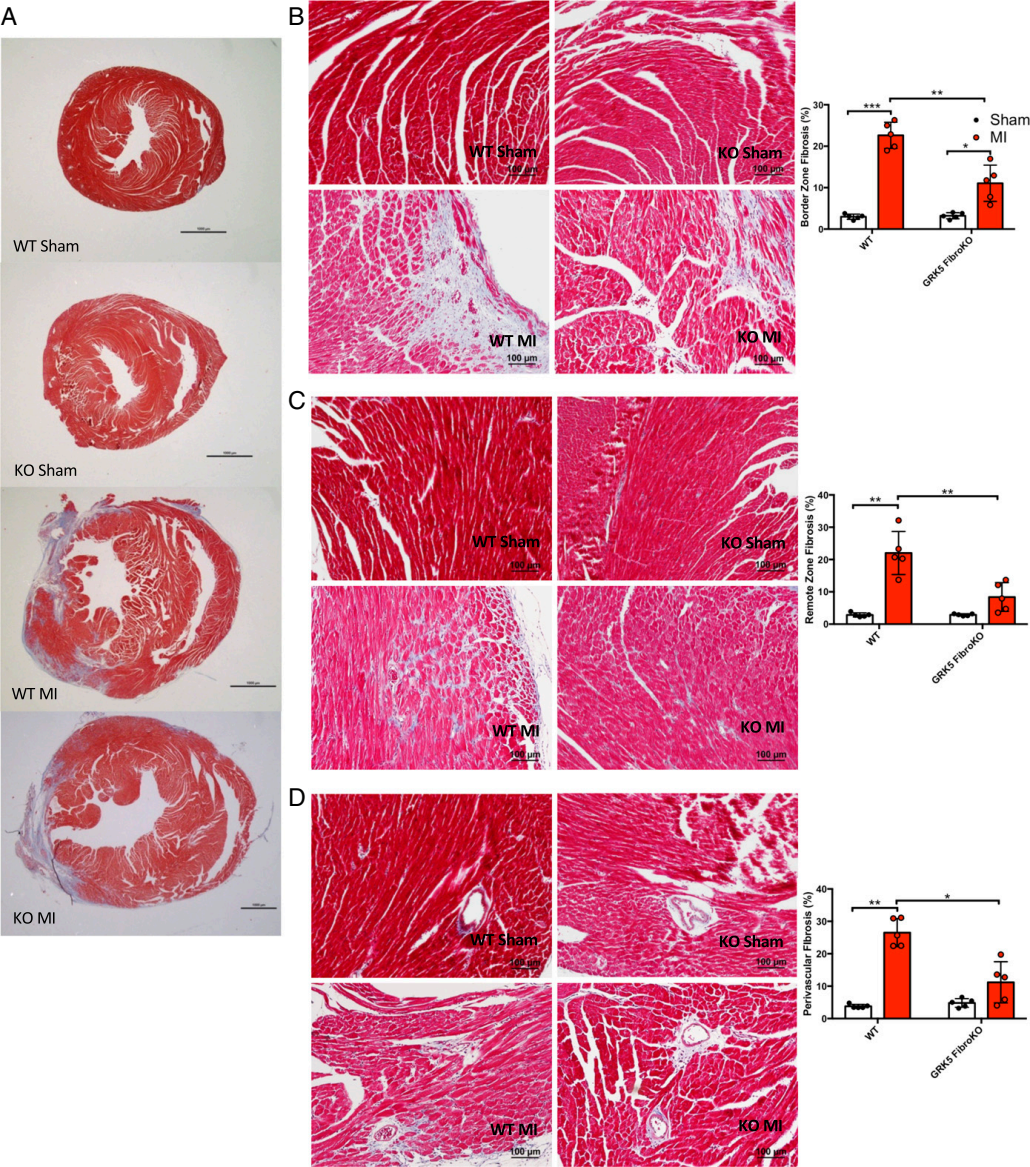
GRK5 fibroKOs are protected against MI mediated cardiac dysfunction and fibrosis. (*A*) Whole heart short-axis images of Masson’s trichrome staining. (*B*–*D*) Representative Masson’s trichrome images and quantification of border zone (*B*), remote zone (*C*), and perivascular (*D*) fibrosis 4 wk after MI surgery. Quantification expressed as a percentage of fibrosis from the total area. *n* = 5 per group. *n* = 5 for sham; 5 to 8 for MI, five images per biological replicate. *P ≤ 0.05, **P ≤ 0.01, ***P ≤ 0.001.

Four weeks after injury, hearts were harvested and heart weights were measured to assess WT and GRK5 fibroKO remodeling after MI. WT mice exhibited cardiac hypertrophy after 4 wk of MI-induced injury compared to sham (Fig. 4*B* and *SI Appendix*, Fig. S7*D*). GRK5 fibroKO hearts were protected against cardiac hypertrophy post-MI (Fig. 4*B* and *SI Appendix*, Fig. S7*D*). Masson’s trichrome staining for fibrosis showed significant fibrosis in the border zone, remote zone, and perivascular region in WT mice 4 wk post-MI, whereas GRK5 fibroKO mice only had a significant increase in fibrosis in the border zone (Fig. 5 *A*–*D*). In all three areas, GRK5 fibroKO hearts displayed a significant decrease in fibrotic area compared to WT mice 4 wk post-MI (Fig. 5 *B*–*D*). Thus, GRK5 in fibroblasts is critical for the fibrotic response in vivo after ischemic injury.

### Noncanonical GRK5 Activity Is Responsible for Fibroblast Activation.

**Fig. 6.**
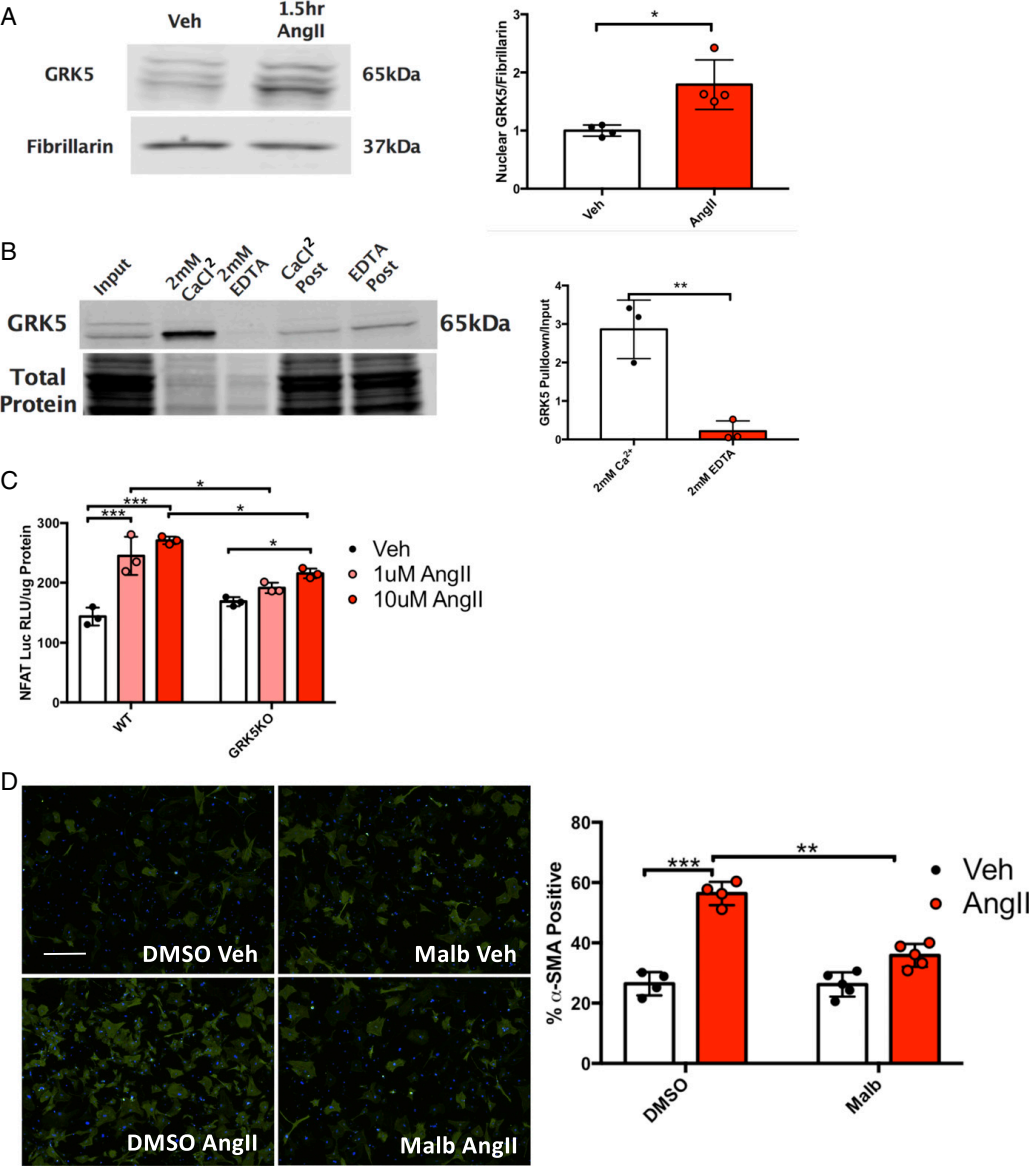
Noncanonical GRK5 activity promotes fibroblast activation. (*A*) Representative immunoblot and quantification of nuclear GRK5 compared to nuclear loading control, fibrillarin, after stimulation of neonatal rat cardiac fibroblasts with AngII (1 µM) for 90 min. *n* = 4. (*B*) Representative immunoblot and quantification of CaM capture. *n* = 3. (*C*) NFAT-luciferase activity from WT and GRK5KO MACFs infected with reporter Ad-NFAT-luc stimulated with AngII. (*D*) Immunofluorescent staining and quantification of α-SMA (green)-positive cells in WT and GRK5KO MACFs stimulated with AngII with or without pretreatment with GRK5 nuclear translocation inhibitor malb. Cells were counterstained with DAPI. *n* = 4 per group. Five images were analyzed per biological replicate. *P ≤ 0.05, **P ≤ 0.01, ***P ≤ 0.001.

GRK5 is a member of the GRK4 subfamily of GRKs, which all contain a nuclear localization signal (NLS) (3, 14, 33). This allows GRK5 to translocate to the nucleus, where it can modulate gene transcription via non-GPCR activity. Previous studies from the W.J.K. laboratory have shown that transgenic (Tg) cardiomyocyte-specific overexpression of GRK5 in mice leads to nuclear and non-GPCR-mediated pathology in the context of pressure-overload-induced HF (15). Mice overexpressing a mutant GRK5 that cannot enter the nucleus (GRK5ΔNLS) were protected against the pathology seen in the TgGRK5 mice emphasizing the importance of nuclear GRK5 in pathology (15). Our studies have shown that GRK5 translocation to the nucleus in myocytes is downstream of Gq activation and binding of Ca^2+^-CaM binding to the N terminus of GRK5 (15, 16). One of the noncanonical activities of GRK5 after its accumulation in the nucleus of myocytes is facilitating hypertrophic gene transcription through activation of NFAT (18). Interestingly, NFAT is critical in myofibroblast transdifferentiation as well (11). In order to see if GRK5 has similar effects in cardiac fibroblasts as in myocytes after injury, neonatal rat cardiac fibroblasts were stimulated with 1 μM AngII for 90 min and nuclear fractions were isolated. There was a significant enrichment of GRK5 in the nucleus after AngII stimulation, suggesting that GRK5 is able to translocate to the nucleus in fibroblasts like in myocytes (Fig. 6*A*). Cytosolic levels of GRK5 remained unchanged (*SI Appendix*, Fig. S9*A*). In order to determine if CaM also binds GRK5 in cardiac fibroblasts, neonatal cardiac fibroblasts were lysed and the lysate was incubated with CaM-conjugated agarose beads in either the presence or absence of Ca^2+^. The CaM beads were able to pull down GRK5 only in the presence of Ca^2+^ (Fig. 6*B*), suggesting that GRK5 binding CaM and subsequent translocation is a Ca^2+^-dependent event, consistent with our previous myocyte studies. Finally, CaM signaling as well as GRK5 noncanonical activity can activate NFAT-mediated gene transcription. In order to see if GRK5 facilitates NFAT activity in the activation of cardiac fibroblasts, we infected WT and GRK5KO MACFs with an NFAT-luciferase adenovirus. We then stimulated the MACFs with 1 and 10 μM AngII for 24 h and measured luciferase activity. WT MACFs stimulated with AngII demonstrated a significant increase in NFAT activity at both 1 and 10 μM AngII. GRK5KO MACFs also exhibited a significant increase in NFAT activity at 10 μM; however, there was an attenuation of NFAT activation compared to WT (Fig. 6*C*). These results suggest that GRK5 is crucial for NFAT-mediated gene transcription during fibroblast activation and this represents a potential contributing mechanism for the amelioration of fibrosis in vivo after chronic AngII treatment and MI above.

We previously reported that malbrancheamide (malb), a fungal indol alkaloid, was able to prevent nuclear translocation of GRK5 in cardiac fibroblasts (26). Furthermore, malb was able to prevent cardiomyocyte hypertrophy after phenylephrine (PE) treatment in vitro (26). To determine if the translocation event is necessary for GRK5’s profibrotic function, we stimulated neonatal cardiac fibroblasts with AngII with or without pretreatment with malb. AngII again led to a significant increase in α-SMA positive cells and interestingly, malb pretreatment significantly impaired fibroblast activation (Fig. 6*D*), implying that nuclear translocation of GRK5 is a critical step required for fibroblast activation.

Because AngII’s profibrotic effects have been demonstrated, in part, due to its induction of TGFβ expression and its subsequent signaling, we investigated the effects of GRK5 deletion in TGFβ-mediated fibroblast activation (34–36). WT and GRK5KO MACFs were treated with TGFβ for 48 h and qRT-PCR was performed. TGFβ stimulation caused an up-regulation of many myofibroblast-associated genes in WT MACFs. However, GRK5KO fibroblasts were only resistant to TGFβ-mediated α-SMA expression, with no differences compared to WT in the other genes measured (*SI Appendix*, Fig. S8*A*). Immunofluorescence staining for α-SMA stress fiber formation was also inhibited in GRK5KO MACFs compared to WT controls after TGFβ treatment (*SI Appendix*, Fig. S8*B*). WT MACFs seeded into a floated collagen matrix demonstrated contraction after AngII stimulation while GRK5KO MACFs had an attenuated contractile response (*SI Appendix*, Fig. S9*B*). Interestingly, TGFβ, a non-GPCR ligand, led to an increase in nuclear translocation of GRK5 (*SI Appendix*, Fig. S8*C*). Because of these results, we wanted to investigate the effects of ET-1, a Gq-coupled ligand which has previously been demonstrated to induce α-SMA expression in fibroblasts and not cause nuclear translocation in cardiomyocytes, on our GRK5KO fibroblasts. ET-1 was able to induce α-SMA expression in both WT and GRK5KO MACFs, suggesting that GRK5 is not involved in ET-1-mediated α-SMA expression (*SI Appendix*, Fig. S9*C*).

## Discussion

Fibroblasts make up a significant proportion of the heart with 11% of the heart composed of fibroblasts compared to 30% for cardiomyocytes (37). In response to cardiac injury, quiescent fibroblasts transdifferentiate into myofibroblast in order to promote healing. However, cardiac myofibroblasts remain active after the healing response and this promotes excessive fibrosis, leading to cardiac remodeling, decreased compliance, and arrhythmogenesis. Treatments for HF that have shown efficacy such as angiotensin converting enzyme (ACE) inhibitors, AngII receptor blockers (ARBs), and mineralocorticoid receptor antagonists have shown that their therapeutic effects are in part due to their ability to decrease the development of fibrosis. However, no therapeutic intervention directly targets the fibrotic response. Targeting nonmyocytes is a potential alternative approach in the treatment of HF, given the critical role myofibroblasts play in the initiation and propagation of cardiac fibrosis and remodeling.

GPCRs comprise roughly 800 human genes and constitute the largest family of proteins targeted by approved drugs (1). Cardiac fibroblasts in particular express 190 of these GPCRs (38). The major modality of GPCR regulation is through GRK activation. GRKs canonically phosphorylate agonist-activated GPCRs to terminate signaling. Because of the sheer number of physiological processes mediated by GPCRs, including angiotensin type 1 receptors, GRKs are crucial in maintaining cardiovascular homeostasis. GRK5 is one of four GRKs expressed in the heart and we demonstrate that it is expressed in cardiac fibroblasts and plays a functional role in fibroblast biology.

The role of GRKs, especially GRK2 and GRK5, has been studied at length in the context of HF. These studies have demonstrated that these critical proteins are important inducers of structural and biochemical changes in the heart. Our group as well as others have investigated the functional role that GRK2 has in fibroblasts in the context of HF (20, 23). Knockout of GRK2 in fibroblasts conferred a protective advantage after myocardial ischemia/reperfusion injury. GRK2 fibroKO mice demonstrated preserved systolic function as well as decreased fibrosis (23). Mechanistically, this was due to a decrease in inflammation. Pharmacological inhibition of GRK2 also was able to prevent fibroblast activation through restoration of β-adrenergic receptor signaling (20). The role of GRK5 in cardiac fibroblasts, however, is not understood. Here we uncovered a previously unknown function of GRK5 in myofibroblast transdifferentiation and cardiac fibrosis in vitro and in vivo. Genetic deletion of GRK5 in cardiac fibroblasts prevented AngII-mediated fibroblast activation. Using an inducible Cre recombinase driven by a fibroblast-specific regulatory sequence, we showed that knockdown of GRK5 in fibroblasts confers a protective advantage for cardiac function and fibrosis in an AngII infusion model as well as an ischemic injury model. These models have widely been utilized to investigate cardiac fibrosis in vivo. Importantly, the AngII infusion model mimics the chronic fibrosis seen in patients with hypertension, whereas our MI model mimics an acute replacement fibrosis followed by a more chronic reactive fibrotic response. In particular, AngII infusion has been utilized as a model of diastolic dysfunction, without changes in systolic function (39–42). While we do not have direct data looking at the diastolic function of our GRK5 fibroKO mice after AngII infusion, we believe that our data demonstrating that GRK5 fibroKO mice are protected against AngII-mediated cardiac hypertrophy and fibrosis suggests that GRK5 inhibition may be beneficial in the context of diastolic dysfunction. Because the signaling pathways underlying these two injuries are distinct, we believe that GRK5 inhibition during the fibrotic response is applicable to a wide variety of cardiac insults.

Recently, the W.J.K. laboratory as well as others, have elucidated noncanonical functions of GRKs and have implicated these in cardiac disease processes. Like other GRKs, GRK5 canonically functions to phosphorylate and inactivate GPCRs and is found on the plasma membrane. However, upon activation of CaM via Ca^2+^-mediated pathways, Ca^2+^-CaM binds GRK5 at both the N and C termini, unhooking it from the plasma membrane (26). GRK5 binds CaM at an affinity 40 times higher compared to GRK2 (43). This binding process decreases GRK5’s affinity for GPCRs while maintaining its ability to phosphorylate and interact with soluble substrates (44, 45). We have shown that nuclear GRK5 in cardiomyocytes can bind directly to DNA as well as phosphorylate histone deacetylase 5 to alter gene transcription after pressure overload (18). Finally, nuclear GRK5 in cardiomyocytes facilitates NFAT-mediated gene transcription during pathological hypertrophy in a transaortic constriction model of HF. GRK5 overexpression increased NFAT activity as shown by utilizing cardiac-specific NFAT-luciferase reporter mice crossed with TK45 mice. Conversely, NFAT-luciferase reporter mice crossed with GRK5KO mice demonstrated diminished NFAT activity after transverse aortic constriction (TAC). Mechanistically GRK5, in a kinase-independent manner, interacts with NFAT at the level of DNA in order to potentiate the binding of the NFAT:DNA complex and promote NFAT-mediated gene transcription (18).

Herein, we show that the same mechanism appears to be intact in cardiac fibroblasts as GRK5KO fibroblasts are resistant to activation after AngII and ischemic stress and have diminished NFAT activity compared to WT fibroblasts. However, NFAT activity is not entirely abolished, which demonstrates that other signaling pathways which feed into NFAT are still active. Furthermore, these data suggest that GRK5 potentiates NFAT-mediated gene transcription but is not absolutely required for its activity. These data are consistent with previous work in cardiac fibroblasts in which activation of NFAT through overexpression of NFAT itself or its upstream activator calcineurin led to activation of fibroblasts (11). Conversely, pharmacological inhibition of NFAT and calcineurin prevented fibroblast conversion (11). In cardiac fibroblasts, NFAT target genes include α-SMA, collagen I, and collagen III as pharmacological inhibition of NFAT leads to the abolishment of transcription of these genes (11, 46). This is in line with our study, as genetic deletion of GRK5 led to diminished NFAT activity and decreased transcription of α-SMA, collagen I, and collagen III. Therefore, we believe that GRK5 is able to induce NFAT-mediated gene transcription and induce myofibroblast-associated gene transcription.

Recent studies indicate that sustained elevation of cytosolic Ca^2+^ can drive the activation of fibroblasts. Neurohormonal signaling molecules which are profibrotic, such as AngII and TGFβ, both trigger an increase in cytosolic Ca^2+^ (47, 48). Furthermore, transient receptor potential (TRP) channels, specifically TRPM7, TRPC6, and TRPV4, which all act to increase cytosolic Ca^2+^ in cardiac fibroblasts, were shown to induce fibroblast transdifferentiation (11, 49, 50). In addition, profibrotic stimuli decrease mitochondrial Ca^2+^ uptake, thereby increasing cytosolic Ca^2+^ (51). Direct inhibition of mitochondrial Ca^2+^ uptake through genetic deletion of Mcu, the pore-forming unit of the mitochondrial Ca^2+^ uniporter, which is responsible for mitochondrial Ca^2+^ uptake, promoted fibroblast activation both at baseline and after profibrotic stimuli (51). Induction of Gαq by AngII leads to an increase in cytosolic Ca^2+^. Past studies in cardiac fibroblasts have shown that 1 μM AngII can increase intracellular Ca^2+^ 24-fold, and that the primary source of this Ca^2+^ was from internal stores (52). This induces Ca^2+^-CaM binding to GRK5, causing dissociation from the plasma membrane and allowing nuclear transport of GRK5, and importantly, we show that this mechanism is active in fibroblasts and contributes to pathology mediated by GRK5. Our studies show that prevention of Ca^2+^-CaM binding to GRK5 with a small molecule, malb, can prevent fibroblast activation and is consistent with the nuclear localization of GRK5-mediating AngII-dependent fibroblast activation and transdifferentiation. Because malb is able to bind CaM, it may alter calcineurin and/or CaM kinase II activation, both of which have been implicated in fibroblast activation (11, 53). Therefore, we cannot solely attribute malb’s effects on fibroblast activation to its inhibition of nuclear GRK5 activity. Importantly, we did not see an increase in GRK5 protein levels after AngII stimulation, implying that translocation is the primary driving event for fibroblast activation rather than its up-regulation.

In both of our AngII and MI models of fibrosis, we saw a reduction in fibrosis that was accompanied by a reduction in the extent of cardiac hypertrophy in GRK5 fibroKO mice. This amelioration of the cardiomyocyte hypertrophic response has also been previously reported in Tgfbr1/2 and Hsp47 deletion in fibroblasts (54, 55). This suggests that activation of fibroblasts and extracellular matrix deposition/myofibroblast-mediated paracrine factors may be required to support the hypertrophic response in cardiomyocytes. Indirect evidence exists suggesting that a stiffer extracellular matrix (ECM) promotes cardiomyocyte hypertrophy; however, further studies are needed to determine the exact mechanism by which myofibroblasts are able to regulate myocyte biology (56). Another interesting result of our study is that loss of fibroblast GRK5 did not alter survival after MI, suggesting no changes in the incidence of cardiac rupture, which can occur when fibrosis and fibroblast activation is severely impaired (11, 54, 57–59). Our results thus indicate that GRK5 is not involved in scar formation and that deletion of fibroblast GRK5 is protective against ischemic injury through modulation of the remodeling response. Scar size was similar at 4 wk, suggesting that fibroblast GRK5 did not alter the acute replacement fibrotic response that occurs immediately after injury. Further, the functional benefits seen in the GRK5 fibroKO mice were only seen 4 wk post MI. Further studies are needed to investigate the role of fibroblast GRK5 in the acute fibrotic response after ischemic injury. In addition, our study demonstrated that GRK5 does not appear to play a significant role in fibroblast proliferation in vitro (*SI Appendix*, Fig. S3). Because fibroblast proliferation is a critical part of the fibrotic response in vivo, the role of fibroblast GRK5 on resident cardiac fibroblast proliferation in vivo utilizing fibroblast-specific reporter mice is of interest (60–62).

**Fig. 7.**
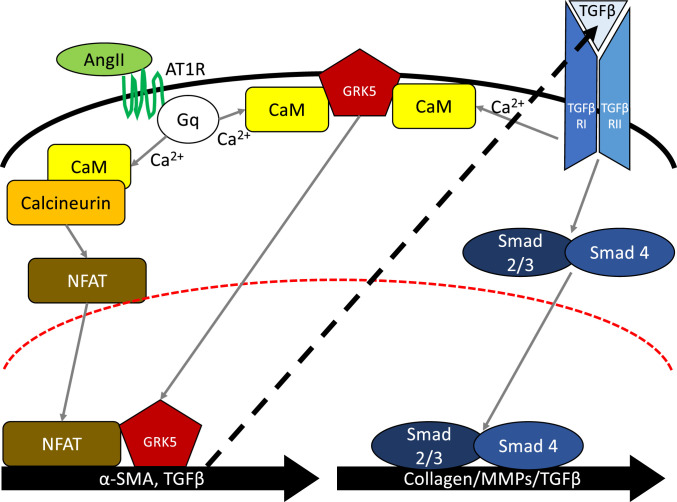
Schematic depicting the signaling cascades for myofibroblast transdifferentiation. AngII signals through activation of NFAT which is potentiated by nuclear GRK5. This leads to expression of α-SMA and TGFβ. TGFβ then acts in an autocrine or paracrine manner activating TGFβ receptors. TGFβ signaling occurs both canonically (Smad mediated) and noncanonically (Ca^2+^) to lead to the expression of myofibroblast-associated genes.

Inhibition of AngII signaling in patients with HF by ACE inhibitors or ARBs have shown to reduce or delay the fibrotic remodeling of the heart (12). AngII is believed to be involved with the fibrotic response both directly and indirectly. Cardiac fibroblasts stimulated with AngII induces TGFβ expression as well as downstream fibrotic genes such as Col1a (34–36). AngII is also able to activate canonical TGFβ signaling through activation of SMADs in cardiac fibroblasts (63). This is in line with our data as AngII stimulation in our WT MACFs caused a significant up-regulation of TGFβ transcript levels while GRK5KO MACFs had significantly reduced TGFβ expression. AngII is also able to directly activate cardiac fibroblasts. AngII stimulation can cause differentiation with or without the presence of TGFβ receptor, suggesting that AngII can independently activate fibroblasts (6). However, inhibition of calcineurin and NFAT downstream of AngII abolished its effects. In our studies, GRK5KO MACFs were resistant to TGFβ-mediated α-SMA expression; however, they had a similar phenotype to WT MACFs with respect to collagen I, collagen III, MMP2, MMP9, and TGFβ mRNA expression. This suggests that there may be two separate signaling axes as illustrated (Fig. 7). AngII is directly able to induce α-SMA expression as well as TGFβ, potentially through GRK5/NFAT activity. TGFβ is then able to cause autocrine or paracrine signaling, leading to its canonical (SMAD mediated) or noncanonical (Ca^2+^ mediated) signaling pathways. TGFβ expression and subsequent signaling appears to be responsible for AngII-mediated expression of Col I, Col III, MMP2, and MMP9 as TGFβ is able to up-regulate these gene in GRK5KO MACFs while GRK5KO MACFs were resistant to AngII-mediated effects on these genes (Fig. 1*A* and *SI Appendix*, Fig. S8*A*). However, GRK5KO MACFs attenuated both AngII- and TGFβ-mediated expression of α-SMA, suggesting that GRK5 has a role in a shared signaling axis. Because GRK5 is able to translocate into the nucleus after both AngII and TGFβ stimulation and NFAT is involved in both signaling cascades, we believe that GRK5 exerts its profibrotic effects through modulating the NFAT response (Fig. 7). Further studies are needed to determine the gene loci that GRK5, NFAT, and Smads bind to in order to tease out the exact mechanisms involved.

Cardiac fibroblasts stimulated with TGFβ have increased GRK5 enrichment in the nucleus, suggesting that nuclear translocation of GRK5 may not entirely be specific to Gq activation. TGFβ has previously been shown to increase cytosolic Ca^2+^, which was prevented by a TGFβ receptor kinase inhibitor (64). Mechanistically, this influx of Ca^2+^ has been demonstrated to be from various sources. Pharmacological inhibitors as well as antibodies against the IP3R prevented TGFβ-induced Ca^2+^ influx, suggesting that this was IP3 mediated at least in mesangial cells (65). Further, TGFβ has been shown to cause release of Ca^2+^ from mitochondria in prostate carcinoma cells (66). In fibroblasts, it appears that TGFβ causes an up-regulation of the mitochondrial Ca^2+^ uptake 1 (MICU1) to increase the threshold needed to allow mitochondrial Ca^2+^ uptake through the mitochondrial Ca^2+^ uniporter. This reduces mitochondrial Ca^2+^ uptake, thereby causing an increase in cytosolic Ca^2+^ (51). GRK5 may be a nodal point in Ca^2+^ signaling and further studies are needed to determine the various physiological activators of GRK5 nuclear translocation.

Because we utilized the Col1a2-cre driver, we cannot rule out the systemic effects of GRK5 knockdown in noncardiac fibroblast populations. Cardiorenal syndrome, a disorder in which cardiac injury, whether acute or chronic, leads to dysfunction in the kidneys has been shown to be a poor prognostic marker for HF patients (67). Inhibition of GRK5 in renal fibroblasts, leading to a preservation of function, may contribute to the phenotype that we see. Because AngII is commonly used to induce renal injury and fibrosis, the mechanism that we observed in cardiac fibroblasts may also be occurring in renal fibroblasts (68, 69). Therefore, a detailed investigation into the role of GRK5 in fibroblasts in other organs such as the kidney, lungs, and liver is important for future analysis.

Therapeutically, our results are significant as they strengthen the argument that GRK5 inhibition is beneficial in the treatment of HF. Previous studies have demonstrated the cardioprotective effects of GRK5 deletion or inhibition in cardiomyocytes in a transaortic constriction model of HF (17, 18). However, there are no data demonstrating the beneficial effects of GRK5 ablation in the fibroblast population. Our goal for this study was to determine the pathophysiological role of fibroblast GRK5 in two models of cardiac fibrosis, one a chronic fibrosis mimicking what is seen in hypertensive patients, and the other an acute replacement fibrotic response followed by a chronic reactive fibrotic response seen in patients after MI. We found that fibroblast GRK5 knockdown inhibits fibroblast activation and the fibrotic response after AngII infusion and MI. While prevention of fibrosis is beneficial, it is not therapeutically applicable in patients. It would be interesting to determine if GRK5 ablation after fibroblast activation has been initiated would be able to reverse the phenotype.

Overall, our work investigated the role of GRK5 in fibroblast activation in vitro and in vivo using two differing models of cardiac fibrosis. We demonstrate that GRK5 is a key regulator of myofibroblast differentiation and its genetic deletion inhibited the fibrotic response. Further, our work provides evidence that the noncanonical roles of GRK5 are pathological and contribute to fibrosis. In conclusion, our work identified a regulator of fibroblast activation and strengthened the argument that GRK5 is a therapeutic target in the treatment of HF.

## Supplementary Material

Supplementary File

## Data Availability

All study data are included in the article and/or supporting information.
