# Supplementary material for: GRK5 is a regulator of fibroblast activation and cardiac fibrosis
Source: Proc Natl Acad Sci U S A. 2021 Jan 26;118(5):e2012854118. doi: 10.1073/pnas.2012854118 (PMC7865138; doi:10.1073/pnas.2012854118)
Supplement: Supplementary File [file pnas.2012854118.sapp.pdf]

SUPPLEMENTAL INFORMATION

DETAILED METHODS

Experimental Animals

To obtain inducible, fibroblast-specific GRK5 KO mice, collagen1α2- CreER(T) mice (Jackson Laboratories Stock no029567) were crossed with GRK5 $^{fl/fl}$ mice (Jackson Laboratories Stock no 010960). Pups were backcrossed to generate collagen1α2-CreER(T)/ GRK5 $^{fl/fl;}$ mice (referred to as GRK5 fibroKO). GRK5 $^{fl/fl}$ mice were used as wild-type (WT) littermate controls. 8-12 week old mice were injected with tamoxifen (Sigma T5648) intraperitoneally for 5 days at 100mg/kg/day. All animal studies were conducted with the approval of the Animal Care and Use Committee at Temple University.

Isolation of Adult and Neonatal Mouse Cardiac Fibroblasts and Myocytes

Hearts were removed from 2 to 3 month old mice and adult cardiac fibroblasts (MACFs) and myocytes were isolated as previously described.[1] Briefly, adult mice were anesthetized with 2% isoflurane and injected with 200 U of heparin 5 min before heart excision. The heart was suspended on a Langendorff apparatus and retrogradely exposed to perfusion buffer (120.4 mM NaCl, 14.7 mM KCl, 0.6 mM KH_2PO_4, 0.6 mM Na_2HPO_4, 1.2 mM $MgSO_4$, 10 mM Hepes, 4.6 mM $NaHCO_3$, 30 mM taurine, 10 mM BDM (2,3-butanedione monoxime), and 5.5 mM glucose) at 37°C. Hearts were then perfused for 12 min with enzyme solution containing 50 mg of collagenase and 0.005% trypsin in perfusion buffer containing 12.5 µM $CaCl_2$. Solution was then switched to perfusion buffer for another 3 min. Atria was removed, and ventricles were gently minced. Cells were dissociated by pipetting in stop solution (perfusion buffer containing 10%

www.pnas.org/cgi/doi/10.1073/pnas.2012854118

fetal bovine serum and 12.5 µM $CaCl_2$). Cardiomyocytes were allowed to settle by centrifugation at 300 rpm for 30 seconds. The supernatant was collected and centrifuged at 500 xg for 5 minutes to collect the first fibroblast (non-myocyte) fraction. Cardiomyocytes were resuspended in 10mL DMEM with 10% FBS and subsequently allowed to settle. Supernatant was collected, centrifuged, and combined with the first fibroblast fraction. Fibroblasts were resuspended and plated in growth media (DMEM with 10% FBS) on a 10cm plate coated with 1% gelatin.

Neonatal rat cardiac fibroblasts (NRCFs) were isolated as a byproduct of neonatal rat cardiac myocyte isolation, performed as previously described.[2] Briefly, hearts were isolated from 1- to 2-day-old neonatal rats. Hearts were pre-washed in ADS buffer (NaCl 116 mM, HEPES 20 mM, Na2HPO4 0.8 mM, glucose 5.6 mM, KCl 7 mM, and MgSO4-7H2O 0.8 mM, pH 7.35) to remove blood and then divided and placed in dishes with 7 ml of ADS. They were minced with sterol razor blades in small pieces and then the whole solutions were transferred in flasks and incubated at 37 °C with 7 ml enzyme solution (ADS containing pancreatin 0.6 mg/L, collagenase II 8820 U/L and CaCl2 50 mM) for 10 min. The supernatant from this pre-digestion step was discarded and the pieces were incubated with 15 ml of digestion solution for 15-min intervals at 37 °C. After each interval, the supernatant was collected in 50 ml conical tubes containing 19 ml of F-10 media and 20% FBS pre-heated at 37 °C. The three to six collected fractions were spun down at 1,400g for 10 min, the supernatant was discarded and cells were washed with 5 ml of FBS for each tube. The cells were then centrifuged at 1,400g for 10 min and the supernatant was discarded. The resulting pellet containing the cells was resuspendend in HAM's F10 complete media containing 10% horse serum (HS), 5% FBS and 1% penicillin-streptomycin (P/S),

pH 7.4. The cell suspension was filtered through a 70 μm filter and pre-plated on a Nunc Nunclon 100 mm (Thermo Fisher Scientific, Waltham, MA, USA) cell culture dish for 2 h to separate the fibroblasts from the myocyte fraction. The fibroblasts attached to the Nunclon dishes were cultured with DMEM with 10% FBS.

Real-time PCR

Total RNA was isolated from MACFs with TRIzol (Thermo Fisher) according to the company's instructions. After RNA isolation, cDNA was synthesized by reverse transcription of the RNA (iScript cDNA synthesis kit, Bio-Rad). Real-time PCR was performed in triplicated on a CFX96 real0time PCR detection system (Bio-Rad) using SYBR Green mix (Bio-Rad) and specific primers for mouse α-SMA, collagen I, collagen III, MMP2, MMP9, and TGFβ. Expression levels were established by comparing to TPT1 expression, which was similar between groups, for normalization and compared using the $\Delta\Delta C_t$ method.

α-SMA: forward 5'-AGATTGTCCGTGACATCAAGG-3'; reverse 5'-TTGTGTGCTAGAGGCAGAGC-3'

Collagen I: forward 5'-ACCTTACCAGACTGAGACTCC-3'; reverse 5'-TTTGGCTCATTCGATCCTGATACCCG-3'

Collagen III: forward 5'-GAAGTCTCTGAAGCTGATGGG-3'; reverse 5'-TTGCCTTGCGTGTTTGATATTC-3'

MMP2: forward 5'-CAGGGAATGAGTACTGGGTCTATT-3'; reverse 5'-ACTCCAGTTAAAGGCAGCATCTAC-3'

MMP9: forward 5'-AATCTCTTCTAGAGACTGGGAAGGAG-3'; reverse 5'-AGCTGATTGACTAAAGTAGCTGGA-3'

TGFβ: forward 5'-CAACAATTCCTGGCGTTACCTTGG-3'; reverse 5'-GAAAGCCCTGTATTCCGTCTCCTT-3'

TPT1: forward 5'-GGAGCTGCAGAGCAGATTAAG -3'; reverse 5'-TAGTCCAGGAGAGCAACCATAC-3'

Surface Sensing of Translation (SUnSET) Assay

WT and GRK5KO MACFs were stimulated with 1μM AngII for varying amounts of time, followed by a pulse of 1μM puromycin for 30 minutes prior to harvesting. Immunoblotting was performed using an anti-puromycin antibody.[3]

Calmodulin Capture Assay

Calmodulin (CaM)-agarose beads (Sigma) were washed with either 2mM $CaCl_2$ or 2mM EDTA in IP buffer (50mM Tris, 150mM NaCl). 250μg of cell lysates were added to the CaM beads with a final concentration of either 2mM $CaCl_2$ or 2mM EDTA IP buffer and rotated overnight. Beads were washed with respective buffers, resuspended in 2X Laemmli buffer, and analyzed by immunoblotting.[4]

Immunoblotting

After SDS-PAGE and transfer to nitrocellulose membranes, primary antibody incubations were performed overnight at 4°C. Fluorescent secondary antibodies were obtained from Li-Cor. Membranes were scanned with the Odyssey infrared imaging system (LI-COR).[5] GRK5 antibody was from Santa Cruz. α-SMA and Puromycin antibody was from Sigma.

In Vivo AngII Infusion and Model of Myocardial Infarction

AngII (1μg/kg/min) dissolved in PBS was continuously infused subcutaneously into mice via an osmotic minipump (ALZET) for 4 weeks. A control group was infused only with PBS. Mice were anesthetized with isofluorane and pumps were implanted subcutaneously through a sub-

scapular incision which was then closed using 4.0 silk suture (Ethicon). Tissue was collected 4 weeks post infusion.

For our MI model, mice were subjected to permanent ligation of the left main descending coronary artery or a sham surgery as we have described previously and tissue was collected 4 weeks post MI.[6]

Transthoracic Echocardiographic Analysis

Transthoracic 2-dimensional echocardiography was performed blinded as previously described using the Vevo 2100 imaging system.[7]

Assessment of Myocardial Fibrosis and Hypertrophy

Collagen levels were measured using the Masson's Trichrome staining kit (Sigma HT15) without modifications as previously described.[5] Briefly, mice were euthanized 4 weeks after MI or AngII infusion. Hearts were removed and fixed in 4% paraformaldehyde, embedded in paraffin, and cut into long axis sections 6μM in thickness. Sections were deparaffinized and rehydrated. Before treatment with the Trichrome staining kit, sections were incubated at room temperature in Bouin's solution (HT101128) overnight. Images of the fibrosis were taken on a Nikon DS-Ri1 in a blinded manner. For each area of the heart, at least 10 random fields were measured. Images were quantified using CellProfiler, a cell image analysis software, capable of determining fibrotic area in an unbiased manner.[8]

Cardiomyocyte hypertrophy was measured using wheat germ agglutinin (WGA) staining as previously described.[9] Briefly, heart sections were deparaffinized, rehydrated, and washed with phosphate-buffered saline. Sections were then stained with Alexa Fluor 488-conjugated WGA for 1 hour at room temperature in the dark. Sections were washed 3 times for 5 minutes

and mounted using Fluoromount-G mounting media containing DAPI nuclear stain (Southern Biotech).

Total Collagen Detection

Hearts were homogenized in 1mg/mL pepsin in .05M acetic acid and incubated at 4° for 48 hours. Supernatant was collected and collagen concentration was detected with a Sirius Red Collagen Detection Kit (Chondrex) without modifications.

Immunofluorescence

$1x10^6$ MACFs seeded on glass coverslips coated with 1% gelatin were fixed with 4% paraformaldehyde, permeabilized with 0.1% Triton X-100, blocked with 5% BSA, and incubated with an anti-αSMA antibody in 5% BSA. Cells were then incubated with respective secondary antibodies.

Drug Treatments

Application of recombinant AngII (1-10uM), TGFβ (1-10ng/mL), and ET-1 (100nM) for 48hrs was used to induce myofibroblast transdifferentiation. Nuclear translocation inhibitor malbrancheamide (malb) was used at 1μM 24 hours before AngII treatments.[10]

NFAT Luciferase Reporter and Luciferase Assay

Cardiac fibroblasts were infected with an NFAT reporter adenovirus at an MOI of 10. Medium was changed after 24 hours, and 48 hours after infection cells were stimulated with AngII for 24 hours. Cells were lysed and luciferase activity measured.[11]

Collagen Gel Contraction Assay

Fibroblasts were harvested from a confluent monolayer by Trypsin-EDTA digestion, pelleted, and resuspended in serum free DMEM. Fibroblasts were then seeded into collagen

matrices (0.85mg/mL) such that each gel contained 100,000 fibroblasts and cast in 24 well plates. The collagen gels were released from the edges and floating in serum free DMEM with or without AngII. ImageJ software was used to calculate the surface area, which are reported as values normalized to the initial size of the gel.[11]

Statistical Tests

Data are expressed as mean ± standard deviation. Statistical significance was determined by ANOVA and Tukey's multiple comparisons test for multivariate experiments and t-test for experiments with two groups.

SUPPLEMENTAL REFERENCES

(1) Travers, J. G., Kamal, F. A., Valiente-Alandi, I., Nieman, M. L., Sargent, M. A., Lorenz, J. N., Molkentin, J. D., and Blaxall, B. C. (2017) Pharmacological and Activated Fibroblast Targeting of Gβγ-GRK2 After Myocardial Ischemia Attenuates Heart Failure Progression. *J. Am. Coll. Cardiol. 70*, 958–971.

(2) Brinks, H., Boucher, M., Gao, E., Chuprun, J. K., Pesant, S., Raake, P. W., Huang, Z. M., Wang, X., Qiu, G., Gumpert, A., Harris, D. M., Eckhart, A. D., Most, P., and Koch, W. J. (2010) Level of G protein-coupled receptor kinase-2 determines myocardial ischemia/reperfusion injury via pro- and anti-apoptotic mechanisms. *Circ. Res. 107*, 1140–1149.

(3) Schmidt, E. K., Clavarino, G., Ceppi, M., and Pierre, P. (2009) SUnSET, a nonradioactive method to monitor protein synthesis. *Nat. Methods 6*, 275–277.

(4) Kaleka, K. S., Petersen, A. N., Florence, M. A., and Gerges, N. Z. (2012) Pull-down of calmodulin-binding proteins. *J. Vis. Exp. JoVE*.

(5) Woodall, M. C., Woodall, B. P., Gao, E., Yuan, A., and Koch, W. J. (2016) Cardiac Fibroblast GRK2 Deletion Enhances Contractility and Remodeling Following Ischemia/Reperfusion Injury. *Circ. Res. 119*, 1116–1127.

(6) Gao, E., Lei, Y. H., Shang, X., Huang, Z. M., Zuo, L., Boucher, M., Fan, Q., Chuprun, J. K., Ma, X. L., and Koch, W. J. (2010) A novel and efficient model of coronary artery ligation and myocardial infarction in the mouse. *Circ. Res. 107*, 1445–1453.

(7) Grisanti, L. A., Traynham, C. J., Repas, A. A., Gao, E., Koch, W. J., and Tilley, D. G. (2016) β2-Adrenergic receptor-dependent chemokine receptor 2 expression regulates leukocyte recruitment to the heart following acute injury. *Proc. Natl. Acad. Sci. U. S. A. 113*, 15126–15131.

(8) McQuin, C., Goodman, A., Chernyshev, V., Kamentsky, L., Cimini, B. A., Karhohs, K. W., Doan, M., Ding, L., Rafelski, S. M., Thirstrup, D., Wiegraebe, W., Singh, S., Becker, T., Caicedo, J. C., and Carpenter, A. E. (2018) CellProfiler 3.0: Next-generation image processing for biology. *PLoS Biol. 16*, e2005970.

(9) Schumacher, S. M., Gao, E., Cohen, M., Lieu, M., Chuprun, J. K., and Koch, W. J. (2016) A peptide of the RGS domain of GRK2 binds and inhibits Gα(q) to suppress pathological cardiac hypertrophy and dysfunction. *Sci. Signal. 9*, ra30.

(10) Beyett, T. S., Fraley, A. E., Labudde, E., Patra, D., Coleman, R. C., Eguchi, A., Glukhova, A., Chen, Q., Williams, R. M., Koch, W. J., Sherman, D. H., and Tesmer, J. J. G. (2019) Perturbation of the interactions of calmodulin with GRK5 using a natural product chemical probe. *Proc. Natl. Acad. Sci. U. S. A. 116*, 15895–15900.

(11) Davis, J., Burr, A. R., Davis, G. F., Birnbaumer, L., and Molkentin, J. D. (2012) A TRPC6-dependent pathway for myofibroblast transdifferentiation and wound healing in vivo. *Dev. Cell 23*, 705–715.

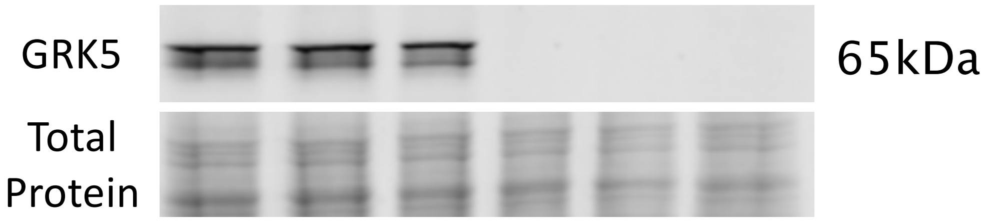

Figure S1. Immunoblot probing for GRK5 in MACFs isolated from WT and GRK5KO MACFs.

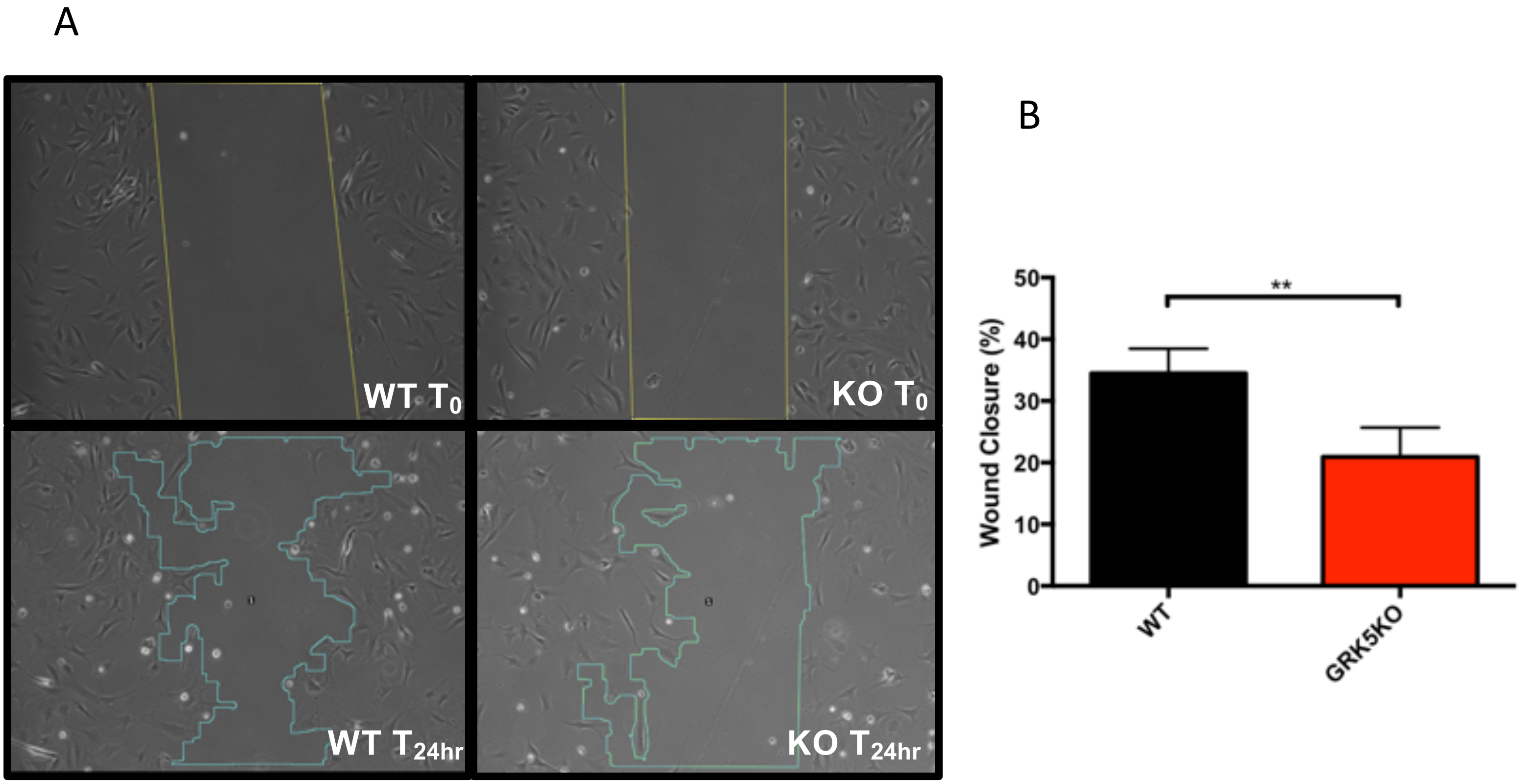

Figure S2. GRK5KO MACFs demonstrate decreased wound closure by *in vitro* scratch assay. Representative image after scratch was initially made and 24 hours after scratch (A) and quantification (B). n = 5 per group. ** $P \leq 0.01$

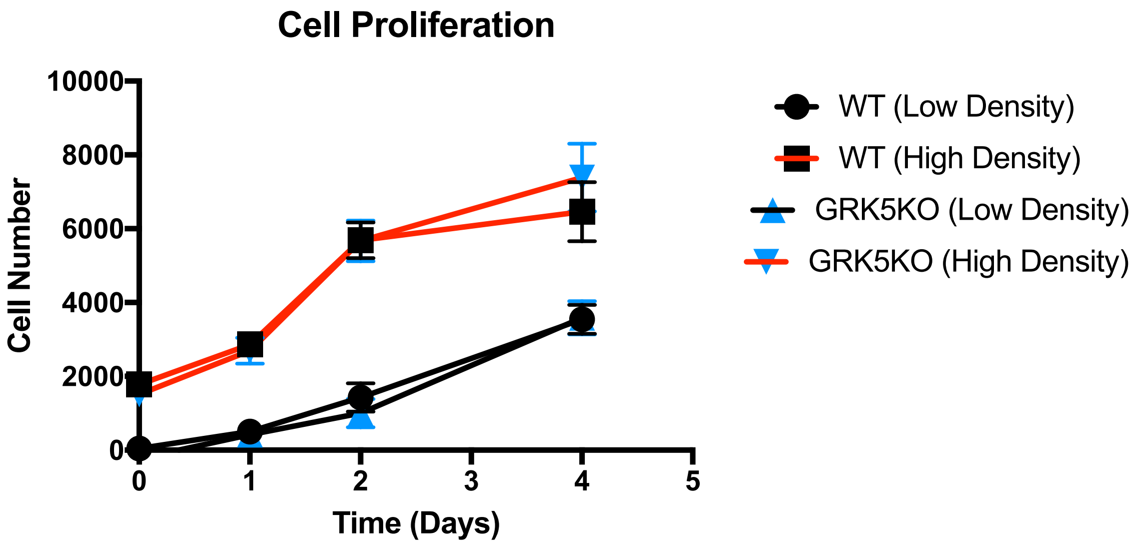

Figure S3. GRK5KO MACFs do not demonstrate changes in proliferation. WT and GRK5KO MACF proliferation measured by CyQUANT cell proliferation assay over 4 days in the presence of 10% FBS. Fibroblasts were initially seeded at either high density (2000 cells/well) or low density (200 cells/well). n = 3 per group.

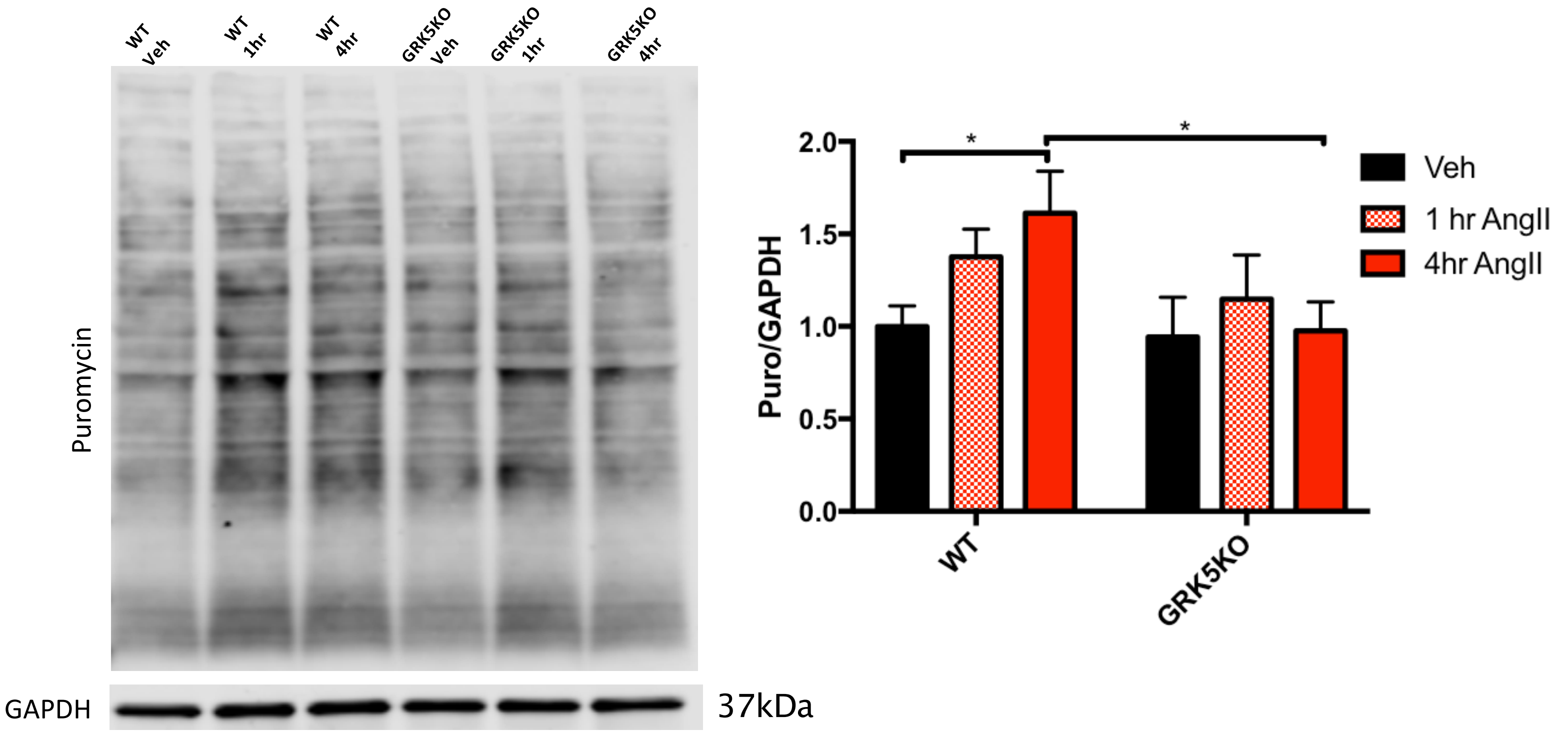

Figure S4. GRK5KO MACFs have decreased protein translation rates after AngII stimulation compared to WT MACFs. Immunoblot and quantification of puromycinylated proteins from MACFs stimulated with 1μM AngII. n = 3 per group. * $P \leq 0.05$

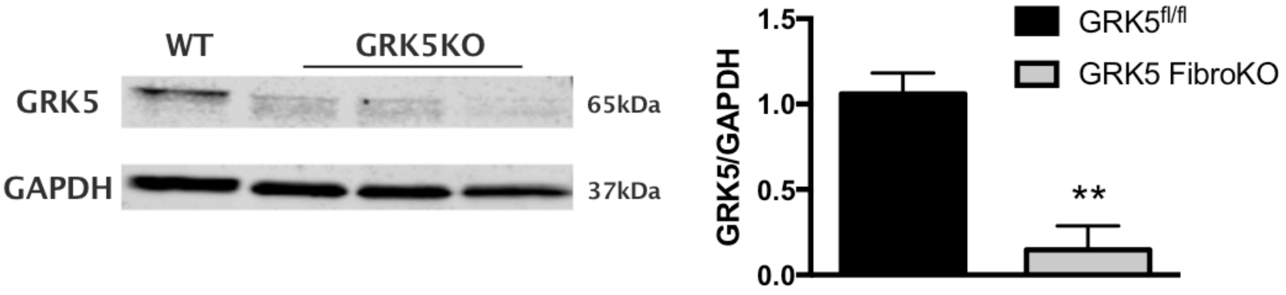

Figure S5. GRK5 levels in fibroblasts isolated from GRK5 fibroKO mice. A) Immunoblot analysis and quantification of GRK5 from MACFs isolated from tamoxifen treated GRK5$^{fl/fl}$ mice or GRK5 FibroKO mice. n = 5 per group ** P ≤ 0.01

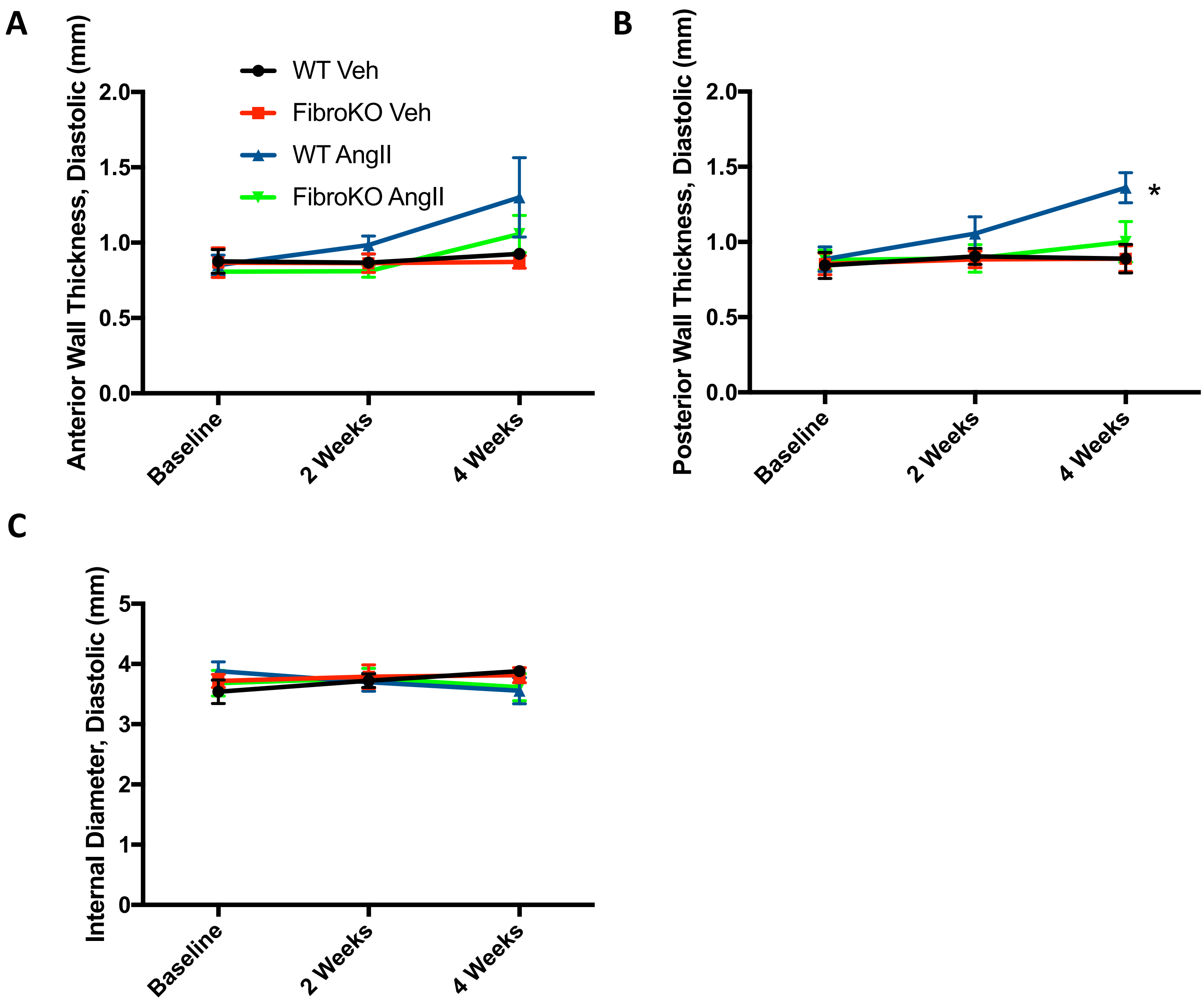

Figure S6. GRK5 fibroKO are protected against AngII mediated cardiac hypertrophy
Echocardiographic analysis of WT and GRK5 fibroKO mice infused with 1µg/kg/min with AngII for 4 weeks compared to 4 weeks of saline infusion. LV wall thickness shown by (A) anterior wall and (B) posterior wall thicknesses at diastole. (C) LV chamber dimentsion shown by internal diameter n = 5 per group * P ≤ 0.05 vs WT Veh

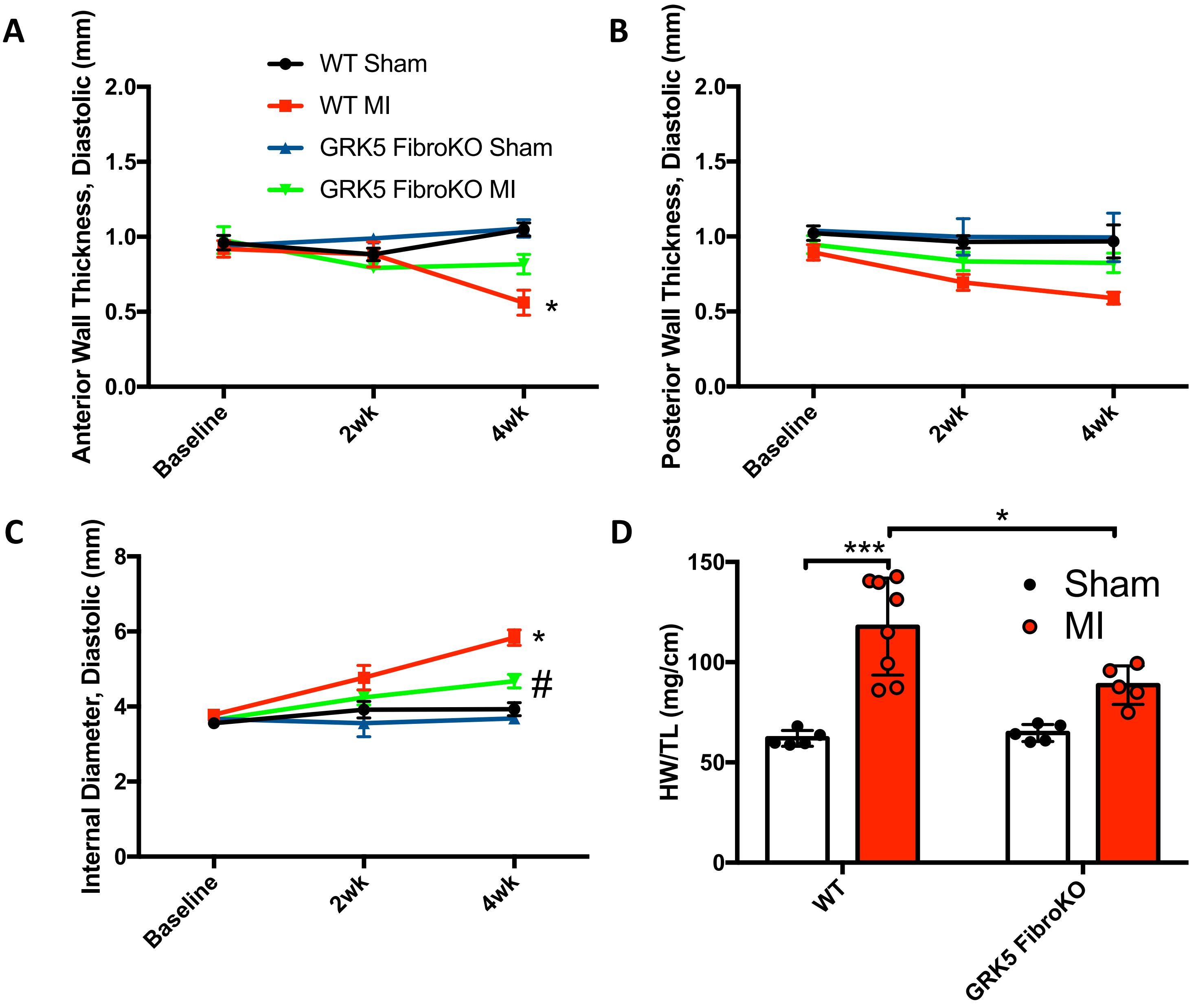

Figure S7. GRK5 fibroKO are protected against MI mediated cardiac dysfunction and hypertrophy. (A) Wall thickness shown by LV anterior wall (LVAW) and (B) posterior wall (LVPW) thicknesses at diastole. (C) LV dilation shown by internal diameter. n = 5 for sham; 5-8 for MI. (D) Quantification of heart weight (HW) normalized to tibia length (TL) 4 weeks after MI surgery. n = 5 for sham; 5-8 for MI.* $P \leq 0.05$ vs Sham, ** $P \leq 0.01$, *** $P \leq 0.001$, # $P \leq 0.05$ vs WT MI

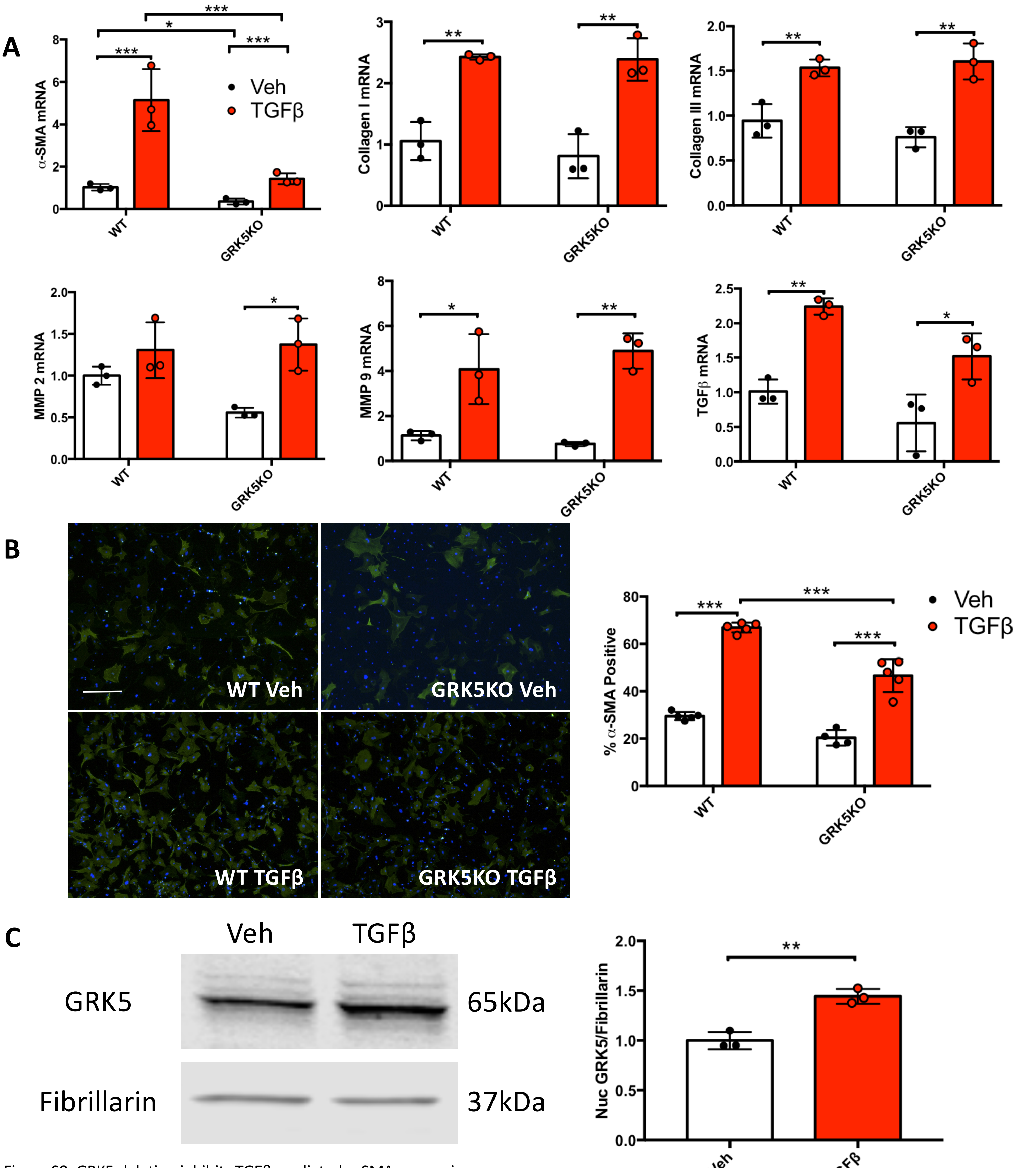

Figure S8. GRK5 deletion inhibits TGFβ mediated α-SMA expression.
(A) Myofibroblast gene expression in WT and GRK5KO MACF's as measured by qRT-PCR normalized to transcriptionally controlled tumor protein 1 (TPT1); fold change vs WT Veh. MACFs were stimulated with 10ng/mL TGFβ for 48 hours. n = 3 per group, 10 images were analyzed per biological replicate. (B) Immunofluorescent staining and quantification of α-SMA (green)-positive cells in WT and GRK5KO MACFs stimulated with AngII. Cells were counterstained with DAPI. n = 3 per group. 5 images were analyzed per biological replicate. (C) Representative immunoblot and quantification of nuclear GRK5 compared to nuclear loading control, fibrillarin, after stimulation of neonatal rat cardiac fibroblasts with TGFβ (10ng/mL) for 30 min. n = 3 .* $P \leq 0.05$, ** $P \leq 0.01$, *** $P \leq 0.001$

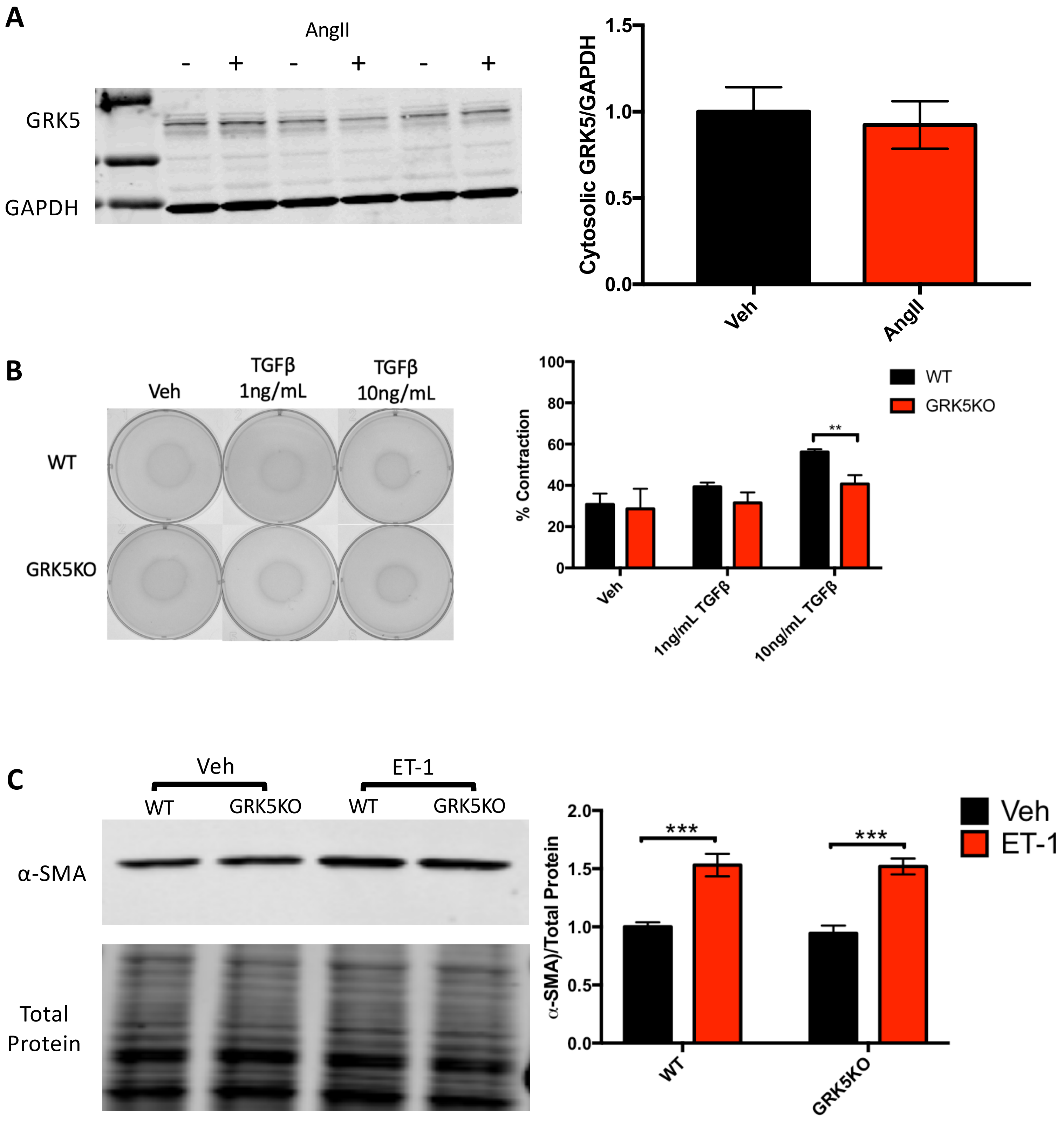

Figure S9. (A) Representative immunoblot and quantification of cytosolic GRK5 compared to cytosolic loading control, GAPDH, after stimulation of neonatal rat cardiac fibroblasts with AngII (1μM) for 90 min. n = 3 (B) Photographs and quantification of floating collagen gel matrices seeded with WT or GRK5KO MACFs that have contracted after 18 hrs of TGFβ stimulation. n = 5 per group. (C) Immunoblot and quantification for α-SMA expression in WT and GRK5KO MACFs stimulated with 100nM ET-1 (normalized to total protein). n = 3 per group * $P \leq 0.05$, ** $P \leq 0.01$, *** $P \leq 0.001$
